# Supplementary material for: Biosynthesis of fragin is controlled by a novel quorum sensing signal
Source: Nat Commun. 2018 Mar 30;9:1297. doi: 10.1038/s41467-018-03690-2 (PMC5878181; doi:10.1038/s41467-018-03690-2)
Supplement: Supplementary file 1 — Supplementary Information(PDF 3947 kb) [file 41467_2018_3690_MOESM1_ESM.pdf]

Biosynthesis of fragin is controlled by a novel quorum sensing signal

Jenul et al.

Supplementary Methods

Synthesis of (–)-(*R*)-Fragin (1)

(9*H*-fluoren-9-yl)methyl (*R*)-(1-azido-3-methylbutan-2-yl)carbamate-(5a)[1]:

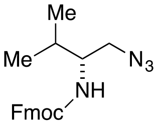

Fmoc-protected amino acid (5.04 g, 14.5 mmol, 1.0 eq) was dissolved in DME (28 ml) and cooled to -15 °C. *N*-Methylmorpholine (1.47 g, 14.5 mmol, 1.0 eq) and isobutyl chloroformiate (2.03 g, 14.5 mmol, 1.0 eq) were added and the mixture stirred at –15 °C for 1.5 h. The suspension was filtered and the filter cake was washed with DME (10 ml). The filtrate was cooled to –15 °C and a solution of $NaBH_4$ (0.787 g, 21.8 mmol, 1.5 eq) in water (7 ml) was added in one portion and the mixture diluted with water (350 ml). The mixture was cooled to 0 °C and the white precipitate was filtered and washed with water (3 times) and pentane (2 times). The white solid was dried at the rotary evaporator (70 °C, 35 mbar) to yield the alcohol **4a** (4.64 g, 14.2 mmol) as a white solid. The obtained data are in good agreement with those reported[1].

The alcohol **4a** (2.64 g, 8.1 mmol, 1.0 eq) and PPh_3 (4.25 g, 16.2 mmol, 2.0 eq) were dissolved in dry THF (68 ml) and cooled to 0 °C. DEAD (7.42 ml, 40% in toluene, 16.2 mmol, 2.0 eq) and then DPPA (4.55 g, 16.2 mmol, 2.0eq) were added and the mixture allowed to warm to RT over 3 h. The solvent was removed after 10 h in total. The crude was purified by flash column chromatography (pentane/EtOAc 6:1) to yield the desired azide **5a** (1.65 g, 4.7 mmol, 57%) as a white solid. The obtained data are in good agreement with those reported[1].

(9*H*-fluoren-9-yl)methyl (*S*)-(1-azido-3-methylbutan-2-yl)carbamate-(5b)[1]:

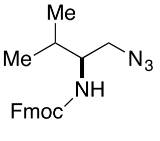

Fmoc-protected amino acid (8.80 g, 25.4 mmol, 1.0 eq) was dissolved in DME (50 ml) and cooled to -15 °C. *N*-Methylmorpholine (2.6 g, 25.4 mmol, 1.0 eq) and isobutyl

chloroformate (3.54 g, 25.4 mmol, 1.0 eq) were added and the mixture stirred at –15 °C for 1.5 h. The suspension was filtered and the filter cake was washed with DME (10 ml). The filtrate was cooled to -15 °C and a solution of $NaBH_4$ (1.47 g, 38.1 mmol, 1.5 eq) in water (13 ml) was added in one portion and the mixture diluted with water (640 ml). The mixture was cooled to 0 °C and the white precipitate was filtered and washed with water (3 x 20 ml) and pentane (2 x 30ml). The white solid was dried at the rotary evaporator (70 °C, 35 mbar) to yield the alcohol **4b** (7.52 g, 23.1 mmol) as a white solid.

The alcohol **4b** (203 mg, 0.62 mmol, 1.0 eq) and PPh_3 (334 mg, 1.25 mmol, 2.0 eq) were dissolved in dry THF (5.3 ml) and cooled to 0 °C. DEAD (0.57 ml, 40% in toluene, 1.25 mmol, 2.0 eq) and then DPPA (350 mg, 1.25 mmol, 2.0eq) were added and the mixture allowed to warm to RT over 3 h. The solvent was removed after a total time of 10 h. The crude was purified by flash column chromatography (pentane/EtOAc 6:1) to yield the desired azide **5b** (152 mg, 0.43 mmol, 63%) as a white solid; **Optical rotation**: $[\alpha]_D^{25}$= +22.8° (*c* 0.43, $CHCl_3$). The data were in good agreement with those reported[1].

(9H-fluoren-9-yl)methyl (R)-(3-methyl-1-octanamidobutan-2-yl)carbamate (7a):

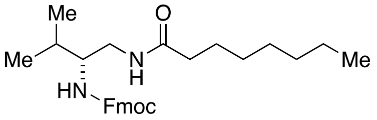

The azide **5a** (1.65 g, 4.71 mmol, 1.0 eq) was dissolved in dry THF/$CHCl_3$ (48ml + 1.3ml, under a hydrogen atmosphere), Pd/C (10%, 501 mg, 0.471 mmol, 0.1 eq) was added and the reaction mixture was stirred at RT. After 12 h, additional Pd/C (10%, 251 mg, 0.236 mmol, 0.05eq) was added, the hydrogen atmosphere renewed and the reaction was stirred for 23 h at RT. The mixture was filtered over Celite and washed with MeOH and the filtrate evaporated. The compound was crystallized by dissolving the solids in hot $CHCl_3$ and cooling the solution to RT. The residue was filtered and dried under vacuum to afford the ammonium salt **6a** (0.966 g, 2.68 mmol), which was used in the next step without any further purification.

The ammonium salt **6a** (907 mg, 2.51 mmol, 1.0 eq) was suspended in dry CH_2Cl_2 (9.7 ml) and octanoyl chloride (459 mg, 2.76 mmol, 1.1 eq) was added. The mixture was cooled to 0 °C and DMAP (31.3 mg, 251 µmol, 0.1 eq) and a solution of DIPEA (729 mg, 5.53 mmol, 2.0 eq) in dry CH_2Cl_2 (3.3 ml) were then added. The solution was

stirred for 30 minutes at 0 °C and then allowed to warm to RT and stirred for 14 h. The mixture was diluted with EtOAc and washed with aq. HCl (1 M, 10.0 ml), sat. aq. $NaHCO_3$ (2 times) and brine (10.0 ml). The organic layer was dried over Na_2SO_4, filtered and evaporated to obtain a brownish solid. The extract was recrystallized from cyclohexane (1.3 g/ 80 ml) by refluxing for 15 min and then cooling to RT. The white suspension was filtered and the residue washed with cold cyclohexane (3 times) to afford the desired amide **7a** (870 mg, 1.93 mmol, 44%) as a white solid; **Optical rotation**: $[\alpha]_D^{25}$= +32.1° (*c* 0.53, $CHCl_3$); $\mathbf{R_f}$= 0.52 (SiO_2, pentane/EtOAc 1:1).

(9H-fluoren-9-yl)methyl (S)-(3-methyl-1-octanamidobutan-2-yl)carbamate (7b):

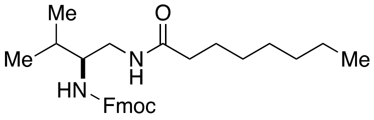

The azide **5b** (2.42 g, 6.92 mmol, 1.0 eq) was dissolved in dry THF/$CHCl_3$ (70 ml + 1.4 ml, under a hydrogen atmosphere), Pd/C (10%, 736 mg, 0.692 mmol 0.1 eq) was added and the reaction mixture was stirred at RT. After 12 h, additional Pd/C (10%, 368 mg, 0.346 mmol, 0.05 eq) was added, the hydrogen atmosphere renewed and the reaction was stirred for 36 h at RT. The mixture was filtered over Celite and washed with MeOH and the filtrate evaporated. The compound was crystallized by dissolving the solids in hot $CHCl_3$ and cooling of the solution cooled to RT. The residue was filtered and dried under vacuum to afford the ammonium salt **6b** (1.60 g, 4.43 mmol), which was used in the next step without any further purification.

The ammonium salt **6b** (200 mg, 0.55 mmol, 1.0 eq) was suspended in dry CH_2Cl_2 (2.0 ml) and octanoyl chloride (101 mg, 0.61 mmol, 1.1 eq) was added. The mixture was cooled to 0 °C and DMAP (6.77 mg, 55.4 µmol, 0.1 eq) and a solution of DIPEA (146 mg, 1.11 mmol, 2.0 eq) in dry CH_2Cl_2 (0.8 ml) were then added. The solution was stirred for 30 minutes at 0 °C and then allowed to warm to RT and stirred for 14 h. The mixture was diluted with EtOAc and washed with aq. HCl (1 M), sat. aq. $NaHCO_3$ (2 times) and brine. The organic layer was dried over Na_2SO_4, filtered and evaporated to dryness to give a brownish solid. The extract was recrystallized from cyclohexane (20 mg/ 3 ml) by refluxing for 15 min and then cooling to RT. The white suspension was filtered and the residue washed with cold cyclohexane (3 times) to afford the desired amide **7b** (198 mg, 0.44 mmol, 51% from **5b**) as a white solid; **Optical rotation**:$[\alpha]_D^{25}$=

–32.6° (*c* 0.20, $CHCl_3$); **M.p.**: 128.3 – 129.0 °C; **FTIR (neat)**:ṽ= 3306, 2953, 2927, 2856, 1690, 1641, 1540, 1450, 1376, 1288, 1252, 1122, 1036, 758, 734, 672 cm^{-1}; **^{1}H NMR** (400 MHz, $CDCl_3$)δ =7.76 (d, *J* = 7.5 Hz, 2H), 7.58 (dd, *J* = 7.2, 4.3 Hz, 2H), 7.40 (t, *J* = 7.4 Hz, 2H), 7.31 (t, *J* = 7.4 Hz, 2H), 6.03 (bs, 1H), 5.02 (d, *J* = 8.9 Hz, 1H), 4.42 (dd,*J* = 10.6, 7.0 Hz 1 H), 4.33 (dd,*J* = 10.5, 7.2 Hz, 1H), 4.20 (t,*J* = 7.0 Hz 1H), 3.61–3.54 (m, 1H), 3.48–3.41 (m, 1H), 3.29 – 3.23 (m, 1H), 2.13 (t, *J* = 7.7 Hz, 2H), 1.81 (sext, *J* = 6.5 Hz, 1H), 1.59 – 1.52 (m, 2H), 1.25 – 1.19 (m, 8H), 0.96 (t, *J* = 7.1 Hz, 6H), 0.83 (t, *J* = 6.7 Hz, 3H); **^{13}C NMR**: (101 MHz, $CDCl_3$) δ = 174.3, 157.5, 144.0, 143.9, 141.4 (2C), 127.9, 127.9, 127.2 (2C), 125.2, 125.1, 120.1, 120,1. 67.0, 57.1, 47.4, 42.4, 36.9, 31.8, 30.9, 29.4, 29.1, 25.9, 22.7, 19.4, 18.3, 14.2; **HRMS (ESI)**: m/z calcd for $C_{28}H_{39}N_2O_3$ $[M+H]^+$: 451.2955, found: 451.2957.

(R)-N-(2-amino-3-methylbutyl)octanamide (8a):

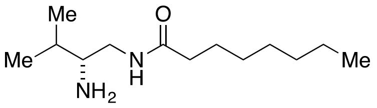

The Fmoc-protected amine **7a** (839 mg, 1.86 mmol, 1.0 eq) was dissolved in dry toluene (36 ml) and $AlCl_3$ (760 mg, 5.59 mmol, 3.0 eq) was added at RT. The yellow solution turned to dark violet over 4 h. An aq. HCl soln. (1 M, 20 ml) was added (pH = 1) and the aq. layer extracted with Et_2O (3 times). A sat. aq. $NaHCO_3$ soln. was added carefully added to the acidic layer. The basic aq. layer (pH = 8) was extracted with CH_2Cl_2 (5 times). The combined organic layers were washed with brine and the organic layer was dried over Na_2SO_4, filtered and evaporated to dryness to afford the desired free amine (403 mg, 1.86 mmol, 95%) as a colorless oil; **Optical rotation**: $[\alpha]_D^{25}$= –34.4° (*c* 0.90, $CHCl_3$); **R_f**= 0.13 (SiO_2, CH_2Cl_2/MeOH 1:1).

(S)-N-(2-amino-3-methylbutyl)octanamide (8b):

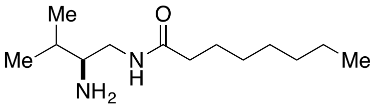

The Fmoc-protected amine **7b** (711 mg, 1.58 mmol, 1.0 eq) was dissolved in dry toluene (31 ml) and $AlCl_3$ (644 mg, 4.73 mmol, 3.0 eq) was added at RT. The yellow solution turned to dark violet over 3.5 h. An aq. HCl soln. (1 M, 20 ml) was added (pH = 1) and the aq. layer extracted with Et_2O (3 times). A sat. aq. $NaHCO_3$soln. (45 ml)

was carefully added to the acidic layer. The basic aq. layer (pH = 8) was extracted with CH_2Cl_2 (5 times). The combined organic layers were washed with brine and the organic layer was dried over Na_2SO_4, filtered and evaporated to dryness to give the desired free amine (330 mg, 1.45 mmol, 92%) as a colorless oil; **Optical rotation**: $[\alpha]_D^{25}$= +41.7° (*c* 0.32, $CHCl_3$); **FTIR (neat)**: ṽ= 3291, 3074, 2956, 2926, 2868, 1642, 1548, 1464, 1369, 1268, 1119, 1029, 671, 629 cm^{-1}; **^{1}H NMR**: (400 MHz, $CDCl_3$) δ =6.16 (bs, 1H), 3.51 (ddd, *J* = 13.4, 6.6, 3.8 Hz, 1H), 2.89 (ddd,*J* = 13.4, 9.3, 4.2 Hz, 1H), 2.56 (ddd,*J* = 9.4, 5.8, 3.8 Hz 1H), 2.18 (t, *J* = 7.5 Hz, 2H), 1.66 – 1.56 (m, 5H), 1.31 – 1.23 (m, 8H), 0.93 (d, *J* = 6.8 Hz, 3H), 0.91 (d, *J* = 6.8 Hz, 3H), 0.86 (t, *J* = 6.9 Hz, 3H); **^{13}C NMR**: (101 MHz, $CDCl_3$) δ = 173.5, 56.8, 43.1, 37.0, 32.6, 31.8, 29.4, 29.2, 26.0, 22.7, 19.3, 17.9, 14.2; **HRMS (ESI)**: m/z calcd for $C_{13}H_{29}N_2O$ $[M+H]^+$: 229.2274, found: 229.2274.

(R)-N-(2-((benzoyloxy)amino)-3-methylbutyl)octanamide (9a):

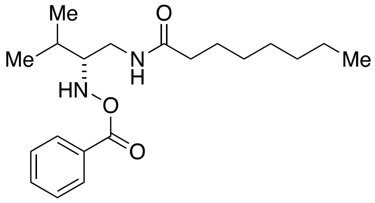

Dibenzoylperoxide (499 mg, 2 mmol, 1.3 eq) and K_2HPO_4 (439 mg, 2.47 mmol, 1.6 eq) were suspended in THF (10 ml). The free amine **8a** (352 mg, 1.54 mmol, 1 eq) was dissolved in THF (4.2 ml) and added to the mixture and stirred for 22.5 h at RT. Eight drops of piperidine were added and the mixture stirred for 10 minutes. Water (5.0 ml) was added and stirring continued at RT until a solution was obtained. The solution was extracted with EtOAc (3 times) and the combined organic layers were washed with brine (2 times), then dried over Na_2SO_4, filtered and evaporated to dryness to afford a yellow oil. The crude was purified by flash column chromatography (SiO_2, pentane/EtOAc 3:1) to give the desired *N*-oxide product **9a** (298 mg, 0.86 mmol, 56%) as a colorless oil; **R_f**= 0.27 (SiO_2, pentane/EtOAc 3:1); **Optical rotation**: $[\alpha]_D^{25}$ = –1.2° (*c* 0.18, $CHCl_3$).

(S)-N-(2-((benzoyloxy)amino)-3-methylbutyl)octanamide (9b):

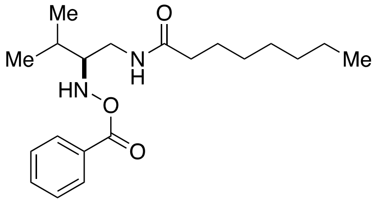

Dibenzoylperoxide (111mg, 0.44 mmol, 1.3 eq) and K_2HPO_4 (97 mg, 0.55 mmol, 1.6 eq) were suspended in THF (2.1 ml). The free amine **8b** (78.1 mg, 0.34 mmol, 1 eq) was dissolved in THF (0.9 ml) and added to the mixture and stirred for 21.5 h at RT. Eight drops of piperidine were added and the mixture stirred for 10 minutes. Water (5 ml) was added and stirring continued at RT until a solution was obtained. The solution was extracted with EtOAc (3 times) and the combined organic layers were washed with brine (2 times), then dried over Na_2SO_4, filtered and evaporated to dryness to afford a yellow oil. The crude was purified by flash column chromatography (SiO_2, pentane/EtOAc 3:1) to give the desired *N*-oxide product **9b** (81.8 mg, 0.24 mmol, 69%) as a colorless oil; **Optical rotation**: $[\alpha]_D^{25}$ = +0.7° (c 0.17, CHCl3); **FTIR (neat):** ṽ = 3289, 3066, 2967, 2927, 2857, 1724, 1643, 1544, 1491, 1452, 1380, 1335, 1263, 1178, 1090, 1025, 982, 858, 786, 707 cm^{-1}; **^{1}H NMR**: (400 MHz, $CDCl_3$) δ = 8.01 – 7.98 (m, 2H), 7.64 – 7.59 (m, 2H), 7.50 – 7.45 (m, 2H); 6.38 (bs, 1H), 3.70 (ddd, *J* = 14.2, 6.0, 3.5 Hz, 1H), 3.29 (ddd,*J* = 13.8, 8.1, 5.1 Hz 1H), 2.96 (td,*J* = 7.2, 3.2 Hz 1H), 2.25 – 2.21 (m, 2H), 1.95 (sext, *J* = 6.7 Hz, 1H), 1.68 – 1.60 (m, 2H), 1.30 – 1.23 (m, 8H), 1.10 (d, *J* = 6.8 Hz, 3H), 1.06 (d, *J* = 6.8 Hz, 3H), 0.87 (t, *J* = 6.9 Hz, 3H); **^{13}C NMR**: (101 MHz, $CDCl_3$) δ = 173.6, 167.0, 133.8, 129.5 (2C), 128.8 (2C), 128.2, 66.7, 38.3, 37.0, 31.8, 29.4, 29.2, 28.3, 25.9, 22.8, 19.5, 19.4, 14.2; **HRMS (ESI)**: m/z calcd for $C_{20}H_{32}N_2O_3$ $[M+H]^+$: 349.2486, found: 349.2490.

(R)-N-(2-(hydroxyamino)-3-methylbutyl)octanamide (10a):

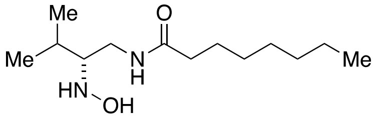

The *N*-oxidized product **9a** (97.7 mg, 280 µmol, 1 eq) was dissolved in dry and degassed EtOH (6.1mL, thaw/freeze method, 3 cycles) and hydrazine monohydrate (0.53 ml, 10.9 mmol, 39.0 eq, 64% in water) was added and the mixture stirred at RT for 2.5 h. The solvent was removed under vacuum and the residue was then suspended in ACN. The suspension was filtered over cotton and then evaporated. The

filtrate was dissolved in Et_2O and aq. HCl soln. (0.25M, 5 ml) and the aq. layer was extracted with Et_2O (3 times). To the acidic aq. layer was added aq. NaOH solution (10 N) until a basic pH was reached. The basic layer was extracted with Et_2O (4 times) and the combined organic layers were dried over Na_2SO_4, filtered and evaporated to dryness to afford the hydroxylamine **10a** (36 mg, 147 µmol, 53%) as a white solid; **R_f**= 0.16 (SiO_2, pentane/EtOAc 1:1); **Optical rotation**: $[\alpha]_D^{25}$= –12.3° (*c* 0.26, $CHCl_3$).

(S)-N-(2-(hydroxyamino)-3-methylbutyl)octanamide (10b):

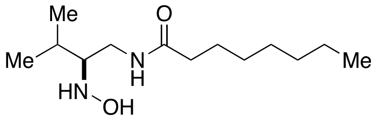

The *N*-oxidized product **9b** (8.2 mg, 23.4 µmol, 1 eq) was dissolved in dry and degassed EtOH (0.5 ml, thaw/freeze method, 3 cycles) and hydrazine monohydrate (0.044 ml, 0.91 mmol, 39.0 eq, 64% in water) was added and the mixture stirred at RT for 2.5 h. The solvent was removed under vacuum and the residue was then suspended in ACN. The suspension was filtered over cotton and then the solvent was evaporated. The filtrate was dissolved in Et_2O (1.5 ml) and aq. HCl soln. (0.25 M,0.5 ml) and the aq. layer was extracted with Et_2O (3 times). To the acidic aq. layer was added aq. NaOH solution (10 N) until a basic pH was reached. The basic layer was extracted with Et_2O (4 times) and the combined organic layers were dried over Na_2SO_4, filtered and evaporated to dryness to afford the hydroxylamine **10b** (3.2 mg, 13 µmol, 56%) as a white solid; **Optical rotation**: $[\alpha]_D^{25}$ = +12.5° (*c* 0.16, $CHCl_3$); **M.p.**: 107.1 – 107.7 °C; **FTIR (neat)**: ṽ= 3311, 3264, 3194, 2950, 2920, 2860, 2852, 1633, 1561, 1466, 1374, 1244, 1163, 1116, 1067, 1003, 912, 832, 721, 671 cm^{-1}; **^{1}H NMR**: (400 MHz, $CDCl_3$) δ = 6.10 (bs, 1H), 5.34 (bs, 2H), 3.60 (ddd,*J* = 14.2, 6.4, 3.4 Hz 1H), 3.29 (ddd,*J* = 14.2, 7.3, 5.7 Hz 1H), 2.53 (dt, *J* = 7.5, 3.4 Hz, 1H), 2.19 (t, *J* = 7.6 Hz, 2H), 1.86 – 1.74 (m, 1H), 1.65 – 1.59 (m, 2H), 1.29 – 1.25 (m, 8H), 0.98 (d, *J* = 6.8 Hz, 3H), 0.95 (d, *J* = 6.8 Hz, 3H), 0.88 – 0.85 (m, 3H); **^{13}C NMR**: (101 MHz, $CDCl_3$) δ = 175.0, 67.2, 38.2, 37.0, 31.8, 29.4, 29.2, 27.5, 25.9, 22.8, 19.7, 19.5, 14.2; **HRMS (ESI)**: m/z calcd for $C_{13}H_{29}N_2O_2$ $[M+H]^+$: 245.2224, found: 245.2226.

(–)-(*R*)-fragin (1):

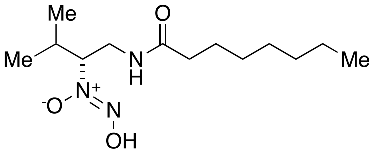

The hydroxylamine **10a** (33.8 mg, 0.14 mmol, 1.0 eq) was dissolved in degassed EtOH (1.4 ml, thaw/freeze method, 3 cycles), isopentyl nitrite (0.13 ml, 0.97 mmol, 7.0 eq) was added and NH_3 gas was bubbled through the solution for two minutes and after 10 minutes again for one minute. After stirring for 30 minutes at RT, the solvent was removed under vacuum and the residue was then extracted with aq. NaOH (1.0 M) and Et_2O (4 times). The basic aq. layer was then acidified with aq. HCl (1.0 M) and extracted with Et_2O (4 times). The combined organic layer was washed with brine, dried over Na_2SO_4, filtered and evaporated to dryness to give (–)-(*R*)-fragin (**1**) (31 mg, 0.12 mmol, 83%) as a white solid; $\mathbf{R}_f$= 0.32 (SiO_2, CH_2Cl_2/MeOH 20:1); **Optical rotation**: $[\alpha]_D^{25}$= –97.7° (*c* 0.54, $CHCl_3$); –134° (*c* 1, EtOH).

(+)-(*S*)-fragin (11)

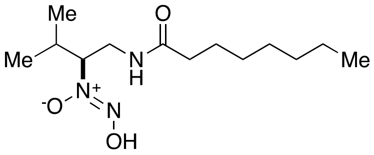

The hydroxylamine **10b** (10.1 mg, 41.5 µmol, 1.0 eq) was dissolved in degassed EtOH (0.4 ml, thaw/freeze method, 3 cycles), isopentyl nitrite (40 µl, 0.29 mmol, 7.0 eq) was added and NH_3 gas was bubbled through the solution for two minutes and after 10 minutes again for one minute. After stirring for 30 minutes at RT, the solvent was removed under vacuum and the residue was then extracted with aq. NaOH (1.0 M) and Et_2O (4 times). The basic aq. layer was then acidified with aq. HCl (1.0 M) and extracted with Et_2O (4 times). The combined organic layer was washed with brine (1 ml), dried over Na_2SO_4, filtered and evaporated to dryness to afford (+)-(*S*)-fragin (**11**) (9.5 mg, 35 µmol, 83%) as a white solid; **Optical rotation**: $[\alpha]_D^{25}$= +126.2° (*c* 0.50, $CHCl_3$); $[\alpha]_D^{25}$= +147.0° (*c* 0.47, EtOH); **M.p.**: 75.2 – 75.9 °C; **FTIR (neat)**: ṽ=3300, 2961, 2926, 2857, 1622, 1566, 1521, 1466, 1424, 1269, 1058, 931, 707 cm^{-1}; **^{1}H NMR**: (400 MHz, $CDCl_3$) δ =11.76 (bs, 1H), 5.68 (bs, 1H), 4.20 (td, *J* = 9.2, 3.1 Hz, 1H), 3.86 (ddd, *J* = 14.4, 6.0, 3.0 Hz, 1H), 3.60 (ddd, *J* = 14.8, 9.4, 6.1 Hz,

1H), 2.25 – 2.17 (m, 1 H), 2.16 – 2.12 (m, 2H), 1.61 – 1.52 (m, 2H), 1.31 – 1.23 (m, 8H), 1.07 (d, *J* = 6.8 Hz, 3H), 0.90 (d, *J* = 6.7 Hz, 3H), 0.87 (t, *J* = 6.8 Hz, 3H); **^{13}C NMR**: (101 MHz, $CDCl_3$)δ = 173.7, 78.0, 39.1, 36.6, 31.8, 29.3, 29.1, 29.1, 25.7, 22.7, 19.1, 18.9, 14.2; **HRMS (ESI)**: m/z calcd for $C_{13}H_{27}N_3O_3Na$ $[M+Na]^+$: 296.1945, found: 296.1945.

Synthesis of the (–)-fragin copper complex:

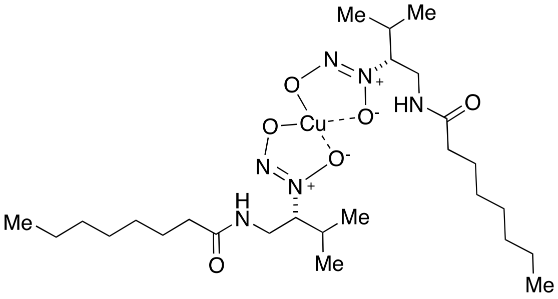

(–)-Fragin (**1**) (2.24 mg, 8.2 µmol, 1.0 eq) was dissolved in dry MeOH and $Cu(OAc)_2$ (2.23 mg, 0.012 mmol, 1.5 eq) was added and the mixture stirred at RT for 18 h. Water was then added and the precipitate filtered over cotton and washed with water (3 times). The filter was flushed with MeOH and the organic layer was dried over $NaSO_4$, filtered and evaporated to dryness to give the Cu-fragin complex (2.27 mg, 7.5 mmol, 91%) as a blue solid. **M.p.**: 154.2 – 154.9 °C; **FTIR (neat)**: ṽ = 3270, 3074, 2959, 2926, 2858, 1638, 1552, 1408, 1465, 1408, 1348, 1277, 1248, 1179, 1124, 998, 934, 861, 796, 709 cm-1; **HRMS (ESI)**: m/z calcd for $C_{26}H_{53}CuN_6O_6$ [M+H]+: 608.3317, found: 608.3316; **UV-VIS**: max = 238 nm.

Synthesis of valdiazen

(*R*)- and (*S*)-2-(hydroxyamino)-3-methylbutan-1-ol (14a and 14b):

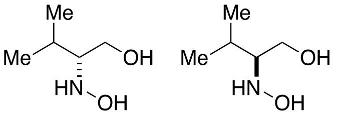

14a **14b**

The hydroxylamines **14a** and **14b** were synthesized according to the literature of Breuning and co-workers[2] and the data were in good agreement.

(–)-(*R*)-valdiazen (12):

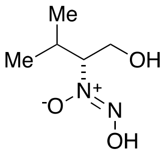

Hydroxylamine **14a** (14.7 mg, 0.123 mmol, 1.0 eq) was dissolved in methanolic ammonia solution (7 N, 0.5 ml) and isopentyl nitrite (43.2 mg, 49.1 ml, 0.369 mmol, 3.0 eq) was added and the mixture stirred at RT. The colorless solution immediately turned yellow. After 5 minutes a white solid precipitated. After 45 min, the solvent was removed under vacuum and the residue dissolved in Et_2O and aq. NaOH (1 M) was added. The aq. layer was extracted with Et_2O (4 times), acidified with aq. HCl (1 M) to pH = 1 and then extracted with Et_2O (4 times). The combined organic layers were washed with brine, dried over $MgSO_4$, filtered and evaporated to dryness to yield (–)-(*R*)-valdiazen (**12**) (8.44 mg, 0.057 mmol, 46%) as a white solid. **M.p.**: 51.4 – 51.9 °C; **Rf** = 0.07 (SiO_2, CH_2Cl_2/MeOH 20:1); **Optical rotation**: $[\alpha]_D^{25}$ = –1.5 (c 0.84, $CHCl_3$); **FTIR (neat)**: ṽ = 3281, 3036, 2974, 2920, 2807, 1549, 1417, 1275, 1253, 1138, 1079, 1043, 1017, 927, 877, 859, 701, 643 cm-1; **^{1}H NMR**: (400 MHz, MeOD) δ = 4.04 – 3.96 (m, 2H), 3.87 – 3.80 (m, 1H), 2.18 – 2.06 (m, 1H), 1.02 (d, J = 6.8 Hz, 3H), 0.90 (d, J = 6.8 Hz, 3H); **^{13}C NMR**: (101 MHz, MeOD) δ = 82.3, 61.2, 29.3, 19.4, 19.4; **HRMS (ESI)**: m/z calcd for $C_5H_{12}N_2O_3Na$ [M+Na]+: 171.07401, found: 171.07378.

(+)-(*S*)-valdiazen (13):

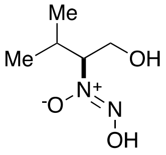

Hydroxylamine **14b** (13.5 mg, 0.113 mmol, 1.0 eq) was dissolved in methanolic ammonia solution (7 N, 0.6 ml) and isopentyl nitrite (45 mL, 0.339 mmol, 3.0 eq) was added and the mixture stirred at RT. The colorless solution immediately turned yellow. After 5 minutes a white solid precipitated. After 45 min, the solvent was removed under vacuum and the residue was dissolved in Et_2O and aq. NaOH (1 M) was added. The aq. layer was extracted with Et_2O (4 times), acidified with aq. HCl (1 M) to pH = 1 and then extracted with Et_2O (4 times). The combined organic layers were washed with brine (1.0 ml), dried over $MgSO_4$, filtered and evaporated to dryness to afford (+)-(*S*)-

valdiazen (**13**) (6 mg, 0.04 mmol, 36%) as a white solid; **Optical rotation**: $[\alpha]_D^{25}$ = +1.0 (*c* 0.60, $CHCl_3$).

Supplementary Notes

Supplementary Note 1: NMR Spectra of (–)-fragin (1)

^{1}H-NMR Spectrum of isolated (–)-fragin (**1**) in $CDCl_3$ (500 MHz)

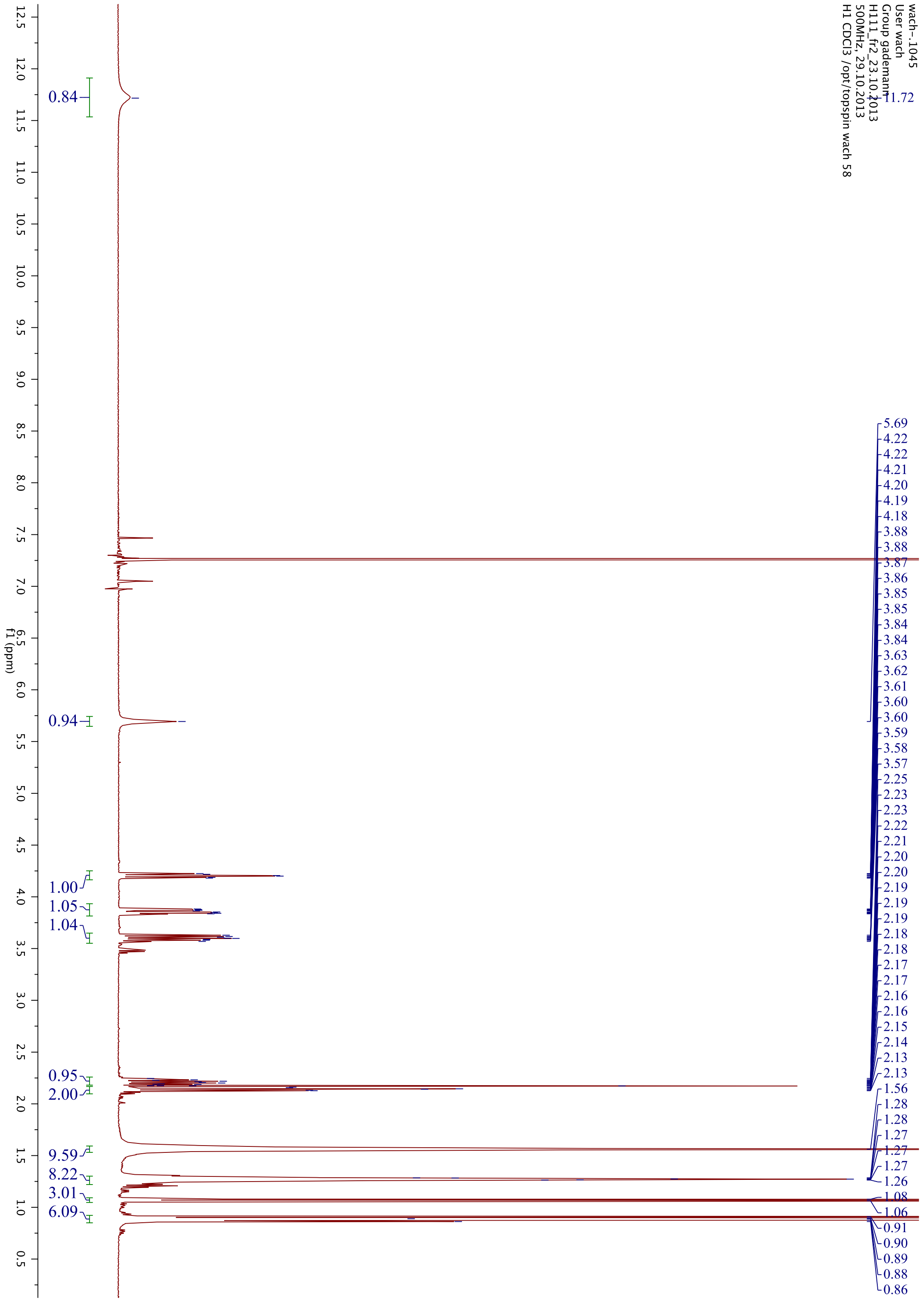

^{1}H-^{1}H COSY NMR Spectrum of isolated (–)-fragin (**1**) in $CDCl_3$ (500 MHz)

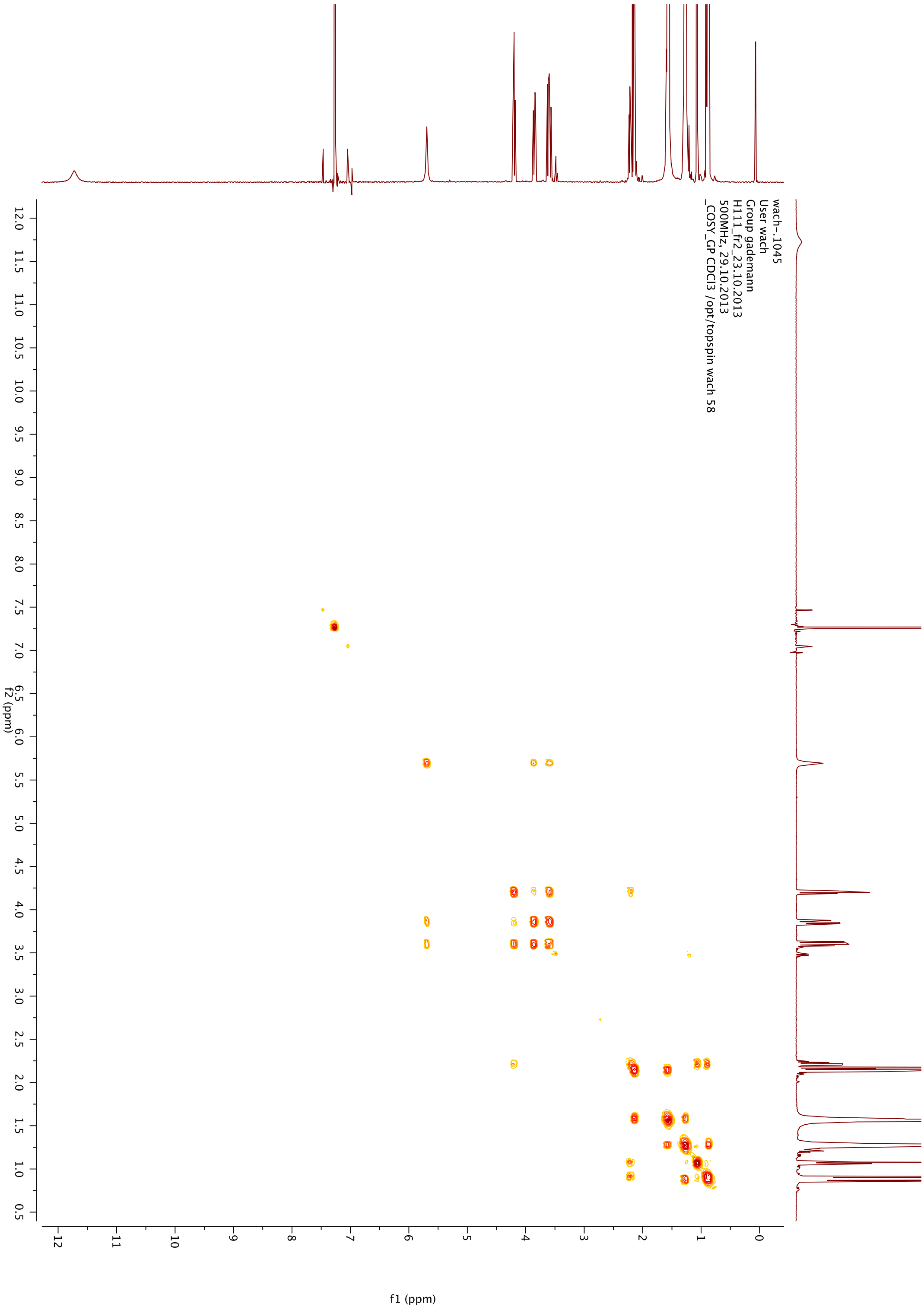

^{1}H-^{13}C HMQC NMR Spectrum of isolated (–)-fragin (**1**) in $CDCl_3$ (500 MHz)

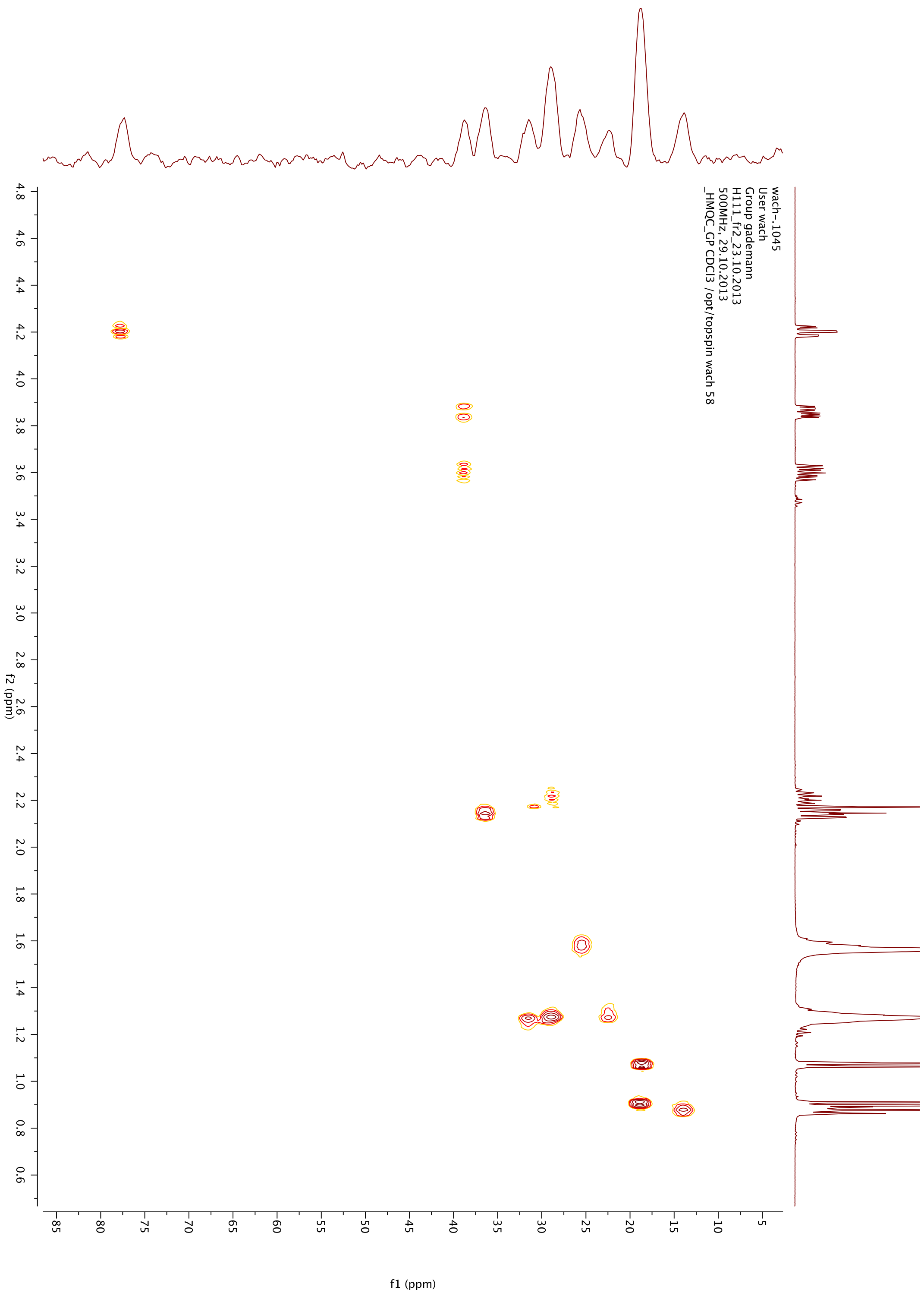

^{1}H NMR Spectrum comparison between isolated (–)-fragin (**1**) and synthetic (–)-fragin (**1**)in $CDCl_3$ (500 MHz)

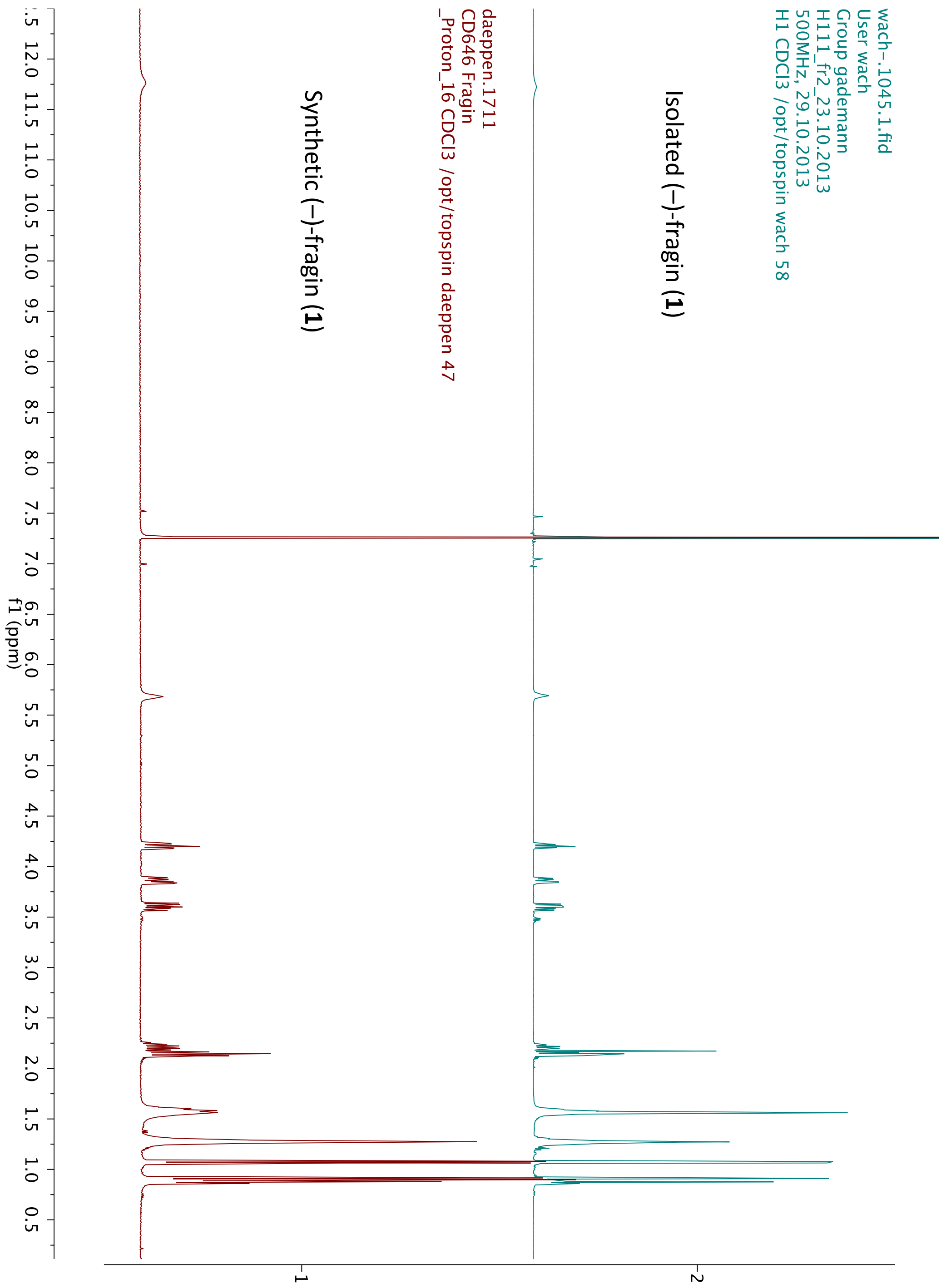

^{13}C NMR Spectrum of the synthetic (+)-fragin (**11**) in $CDCl_3$ (126 MHz)

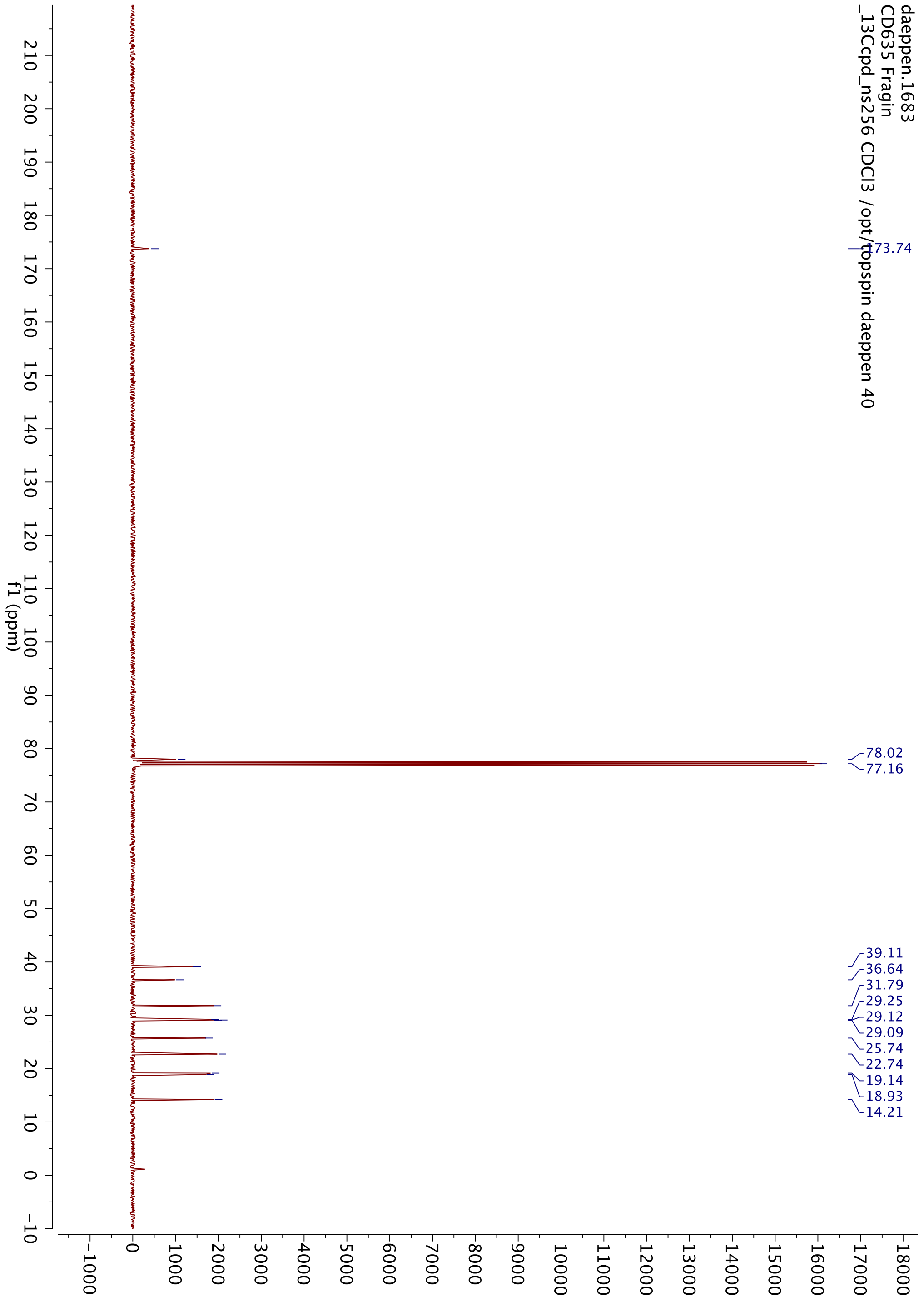

^{1}H-NMR Spectrum of the Fmoc protected **7b** in $CDCl_3$

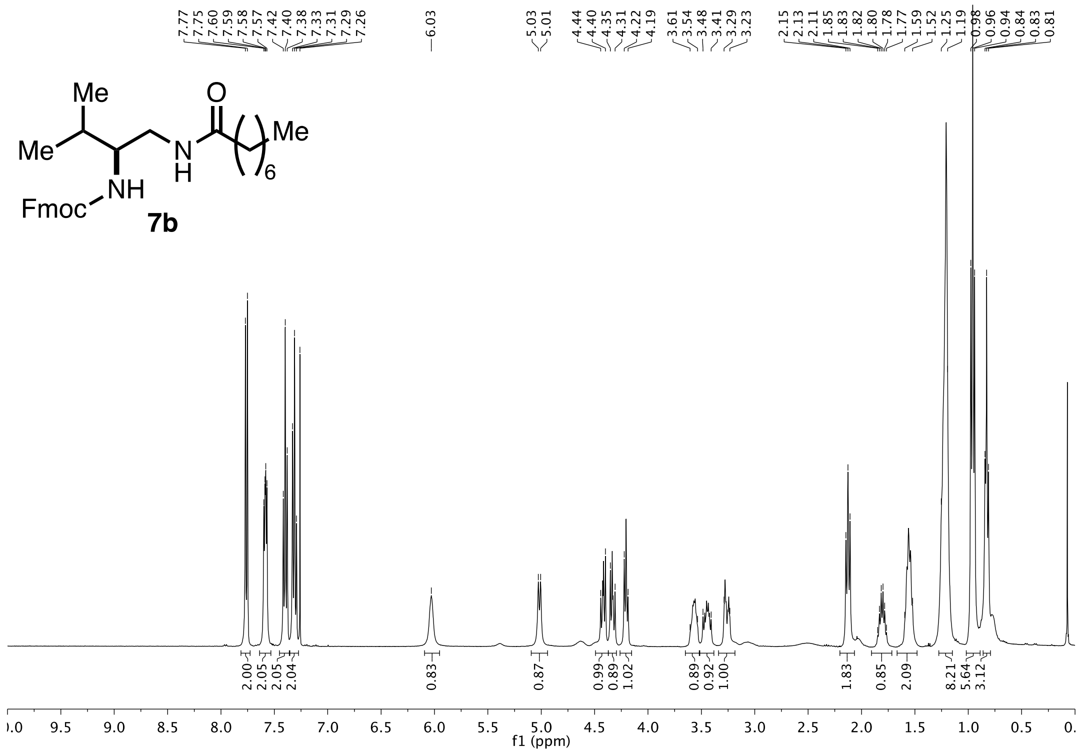

^{13}C-NMR Spectrum of the Fmoc protected **7b** in $CDCl_3$

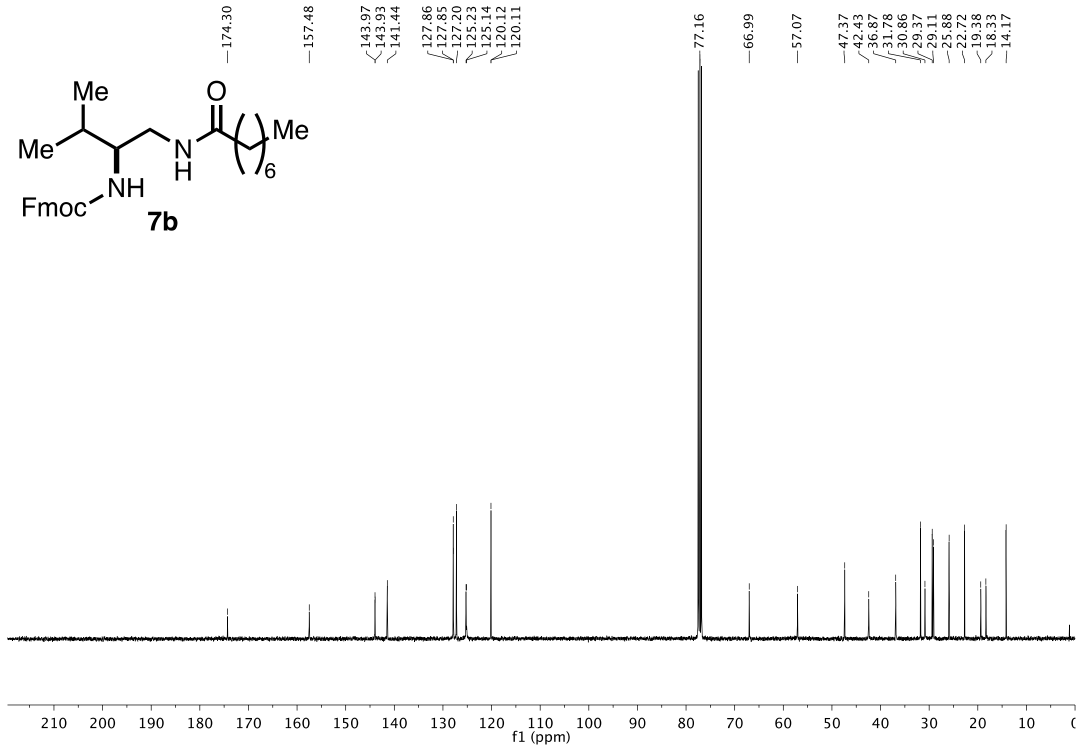

^{1}H-NMR Spectrum of the amine **8b** in CDCl$_3$

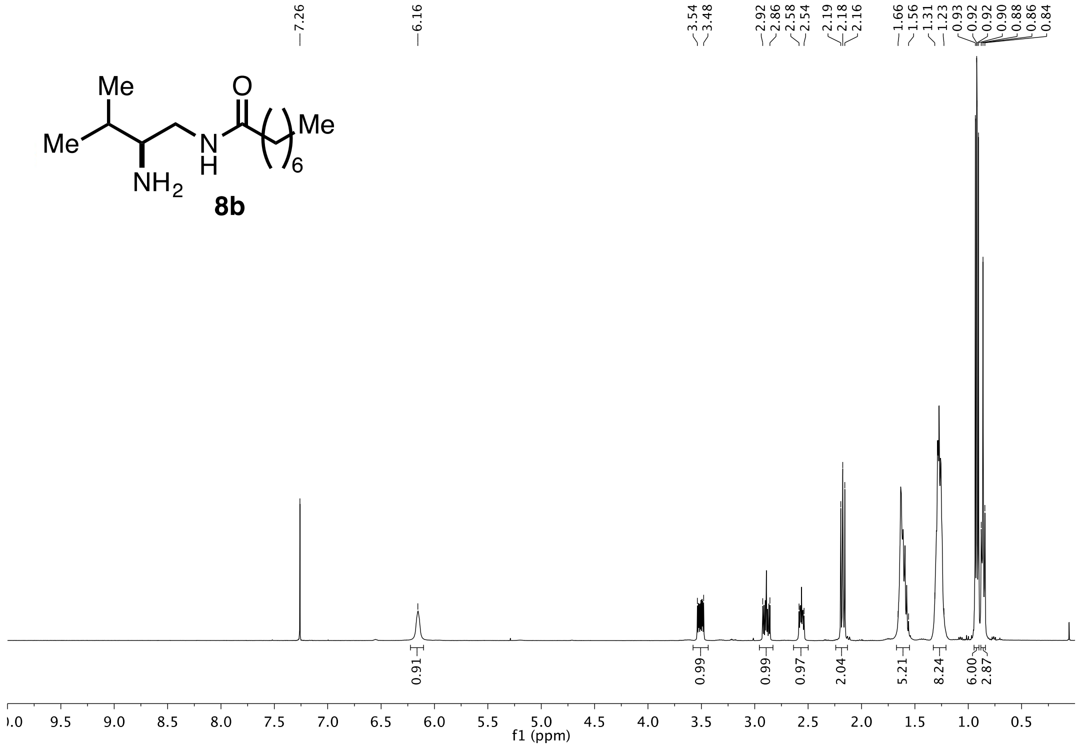

^{13}C-NMR Spectrum of the amine **8b** in CDCl$_3$

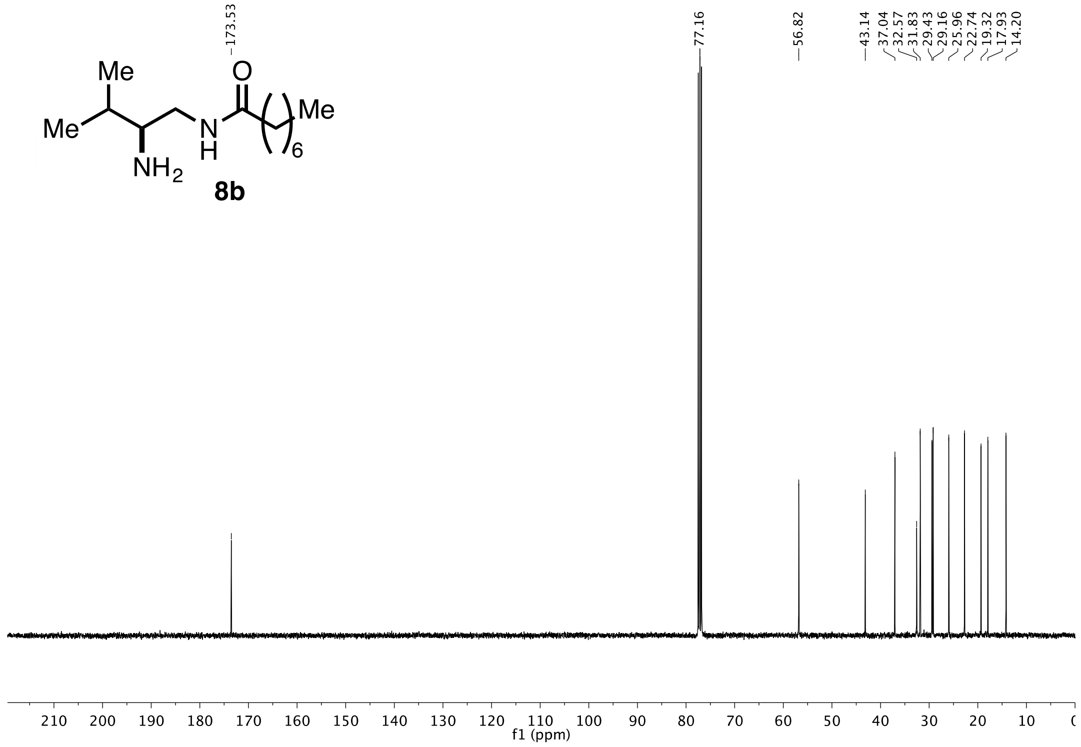

^{1}H-NMR Spectrum of the protected hydroxylamine **9b** in $CDCl_3$

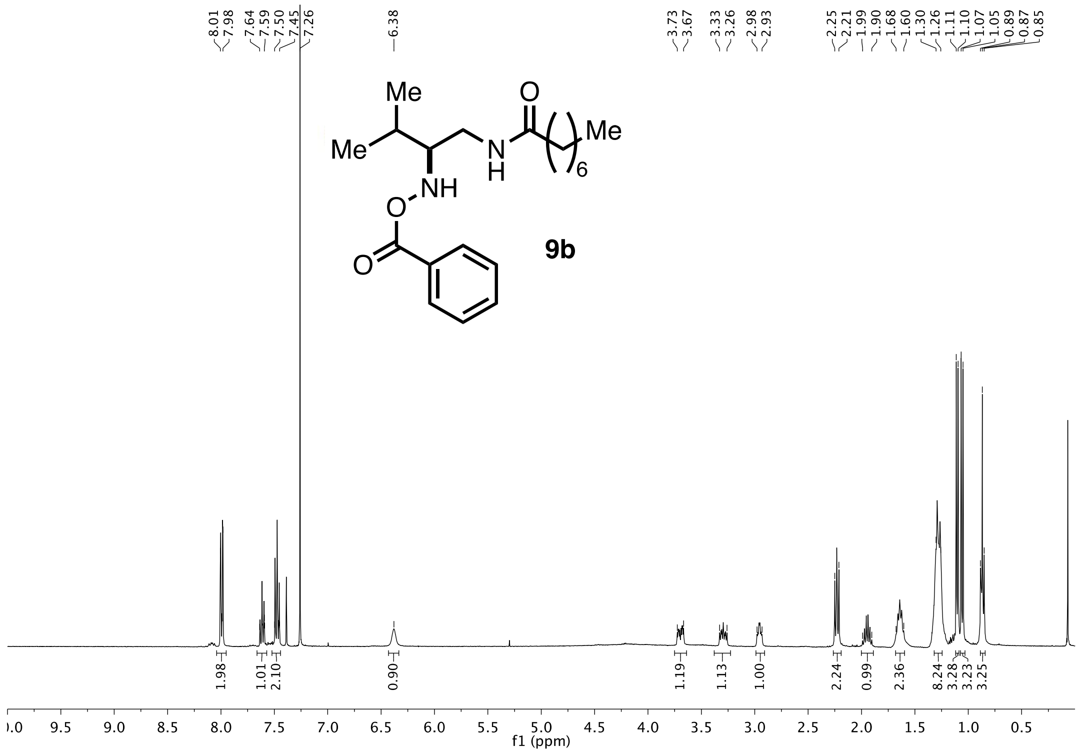

^{13}C-NMR Spectrum of the protected hydroxylamine **9b** in $CDCl_3$

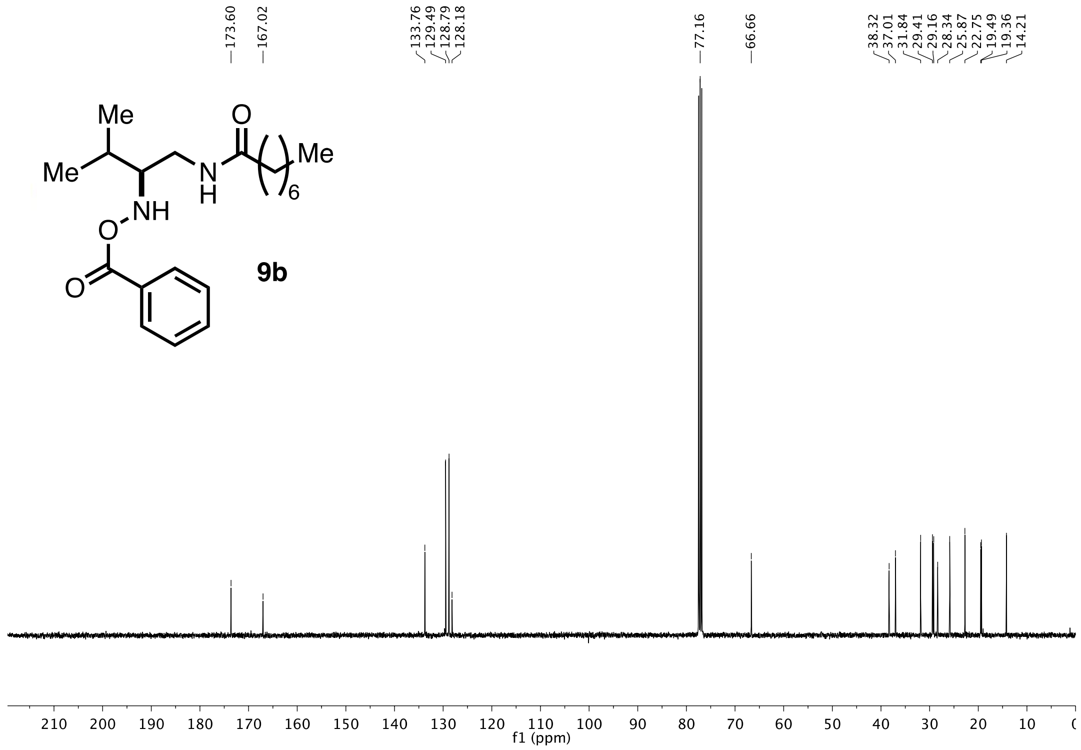

^{1}H-NMR Spectrum of the hydroxylamine **10b** in $CDCl_3$

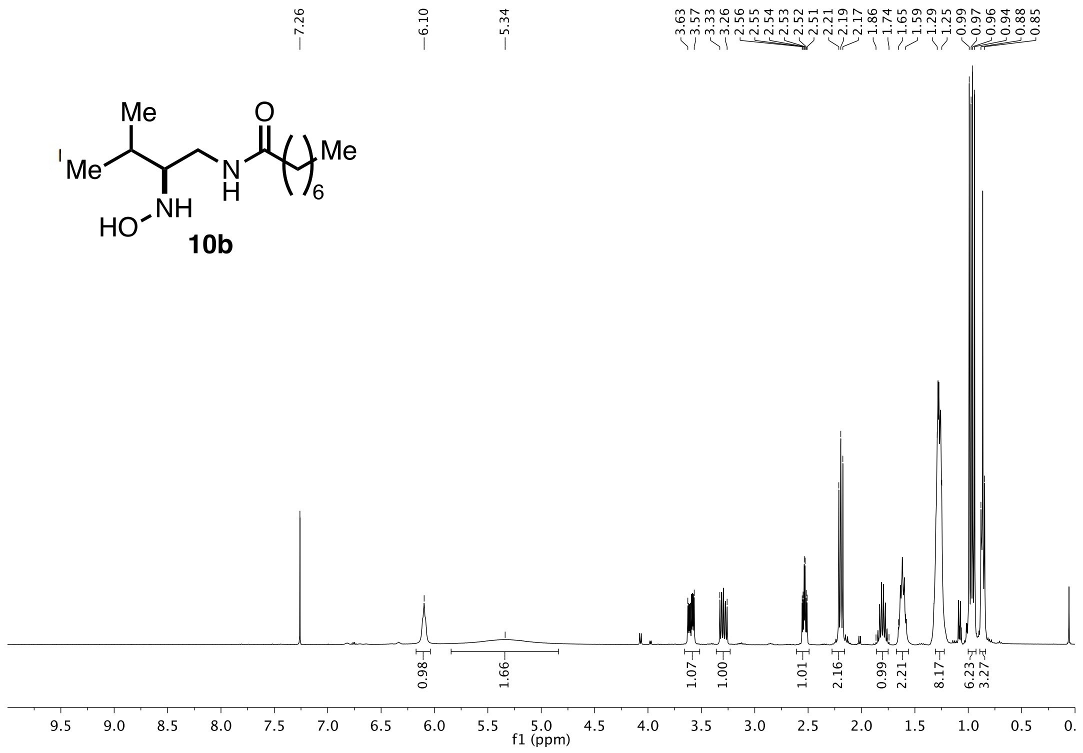

^{13}C-NMR Spectrum of the hydroxylamine **10b** in $CDCl_3$

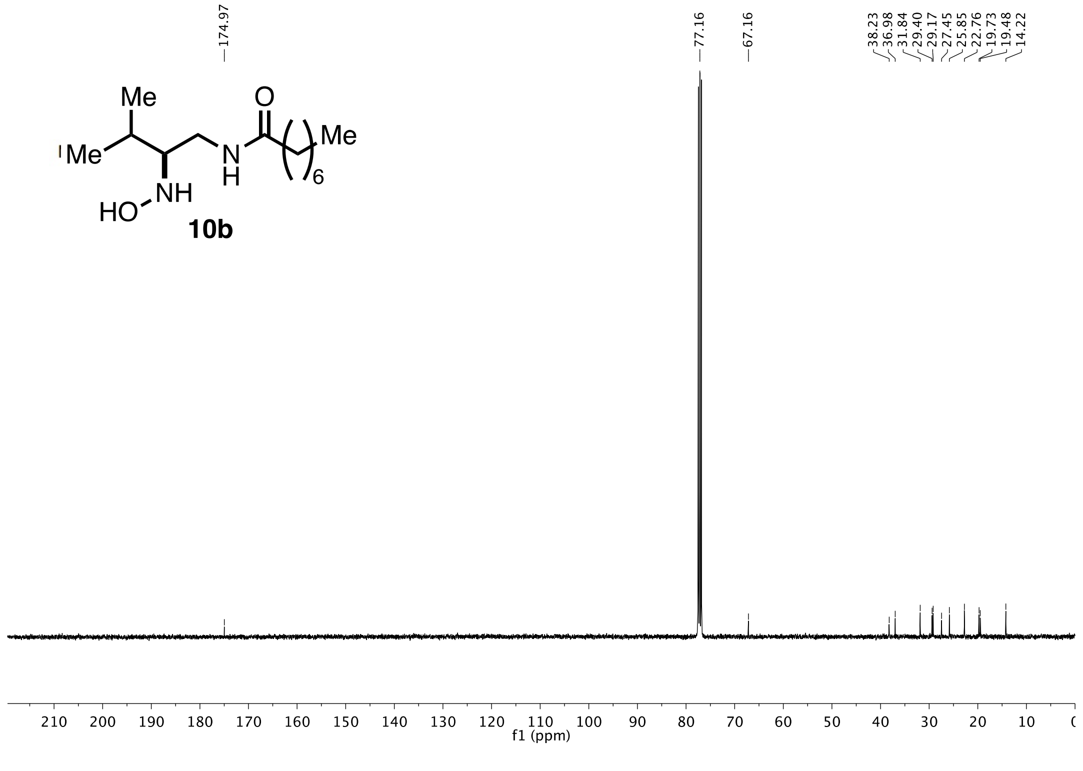

^{1}H-NMR Spectrum of synthetic (–)-fragin (**1**) in $CDCl_3$

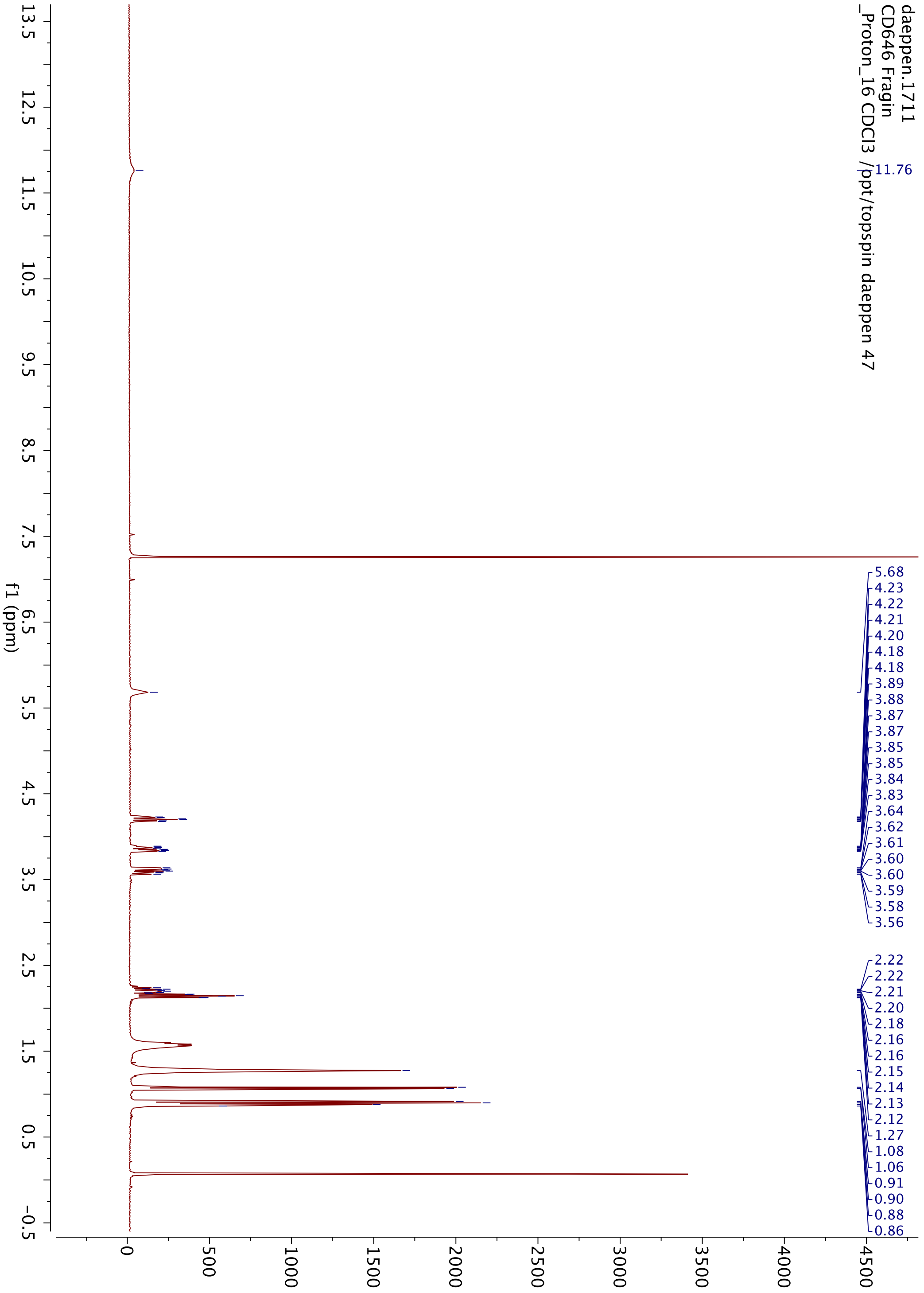

Supplementary Note 2: NMR spectra of valdiazen

^{1}H-NMR Spectrum of isolated valdiazen (**2**) in CD_3OD

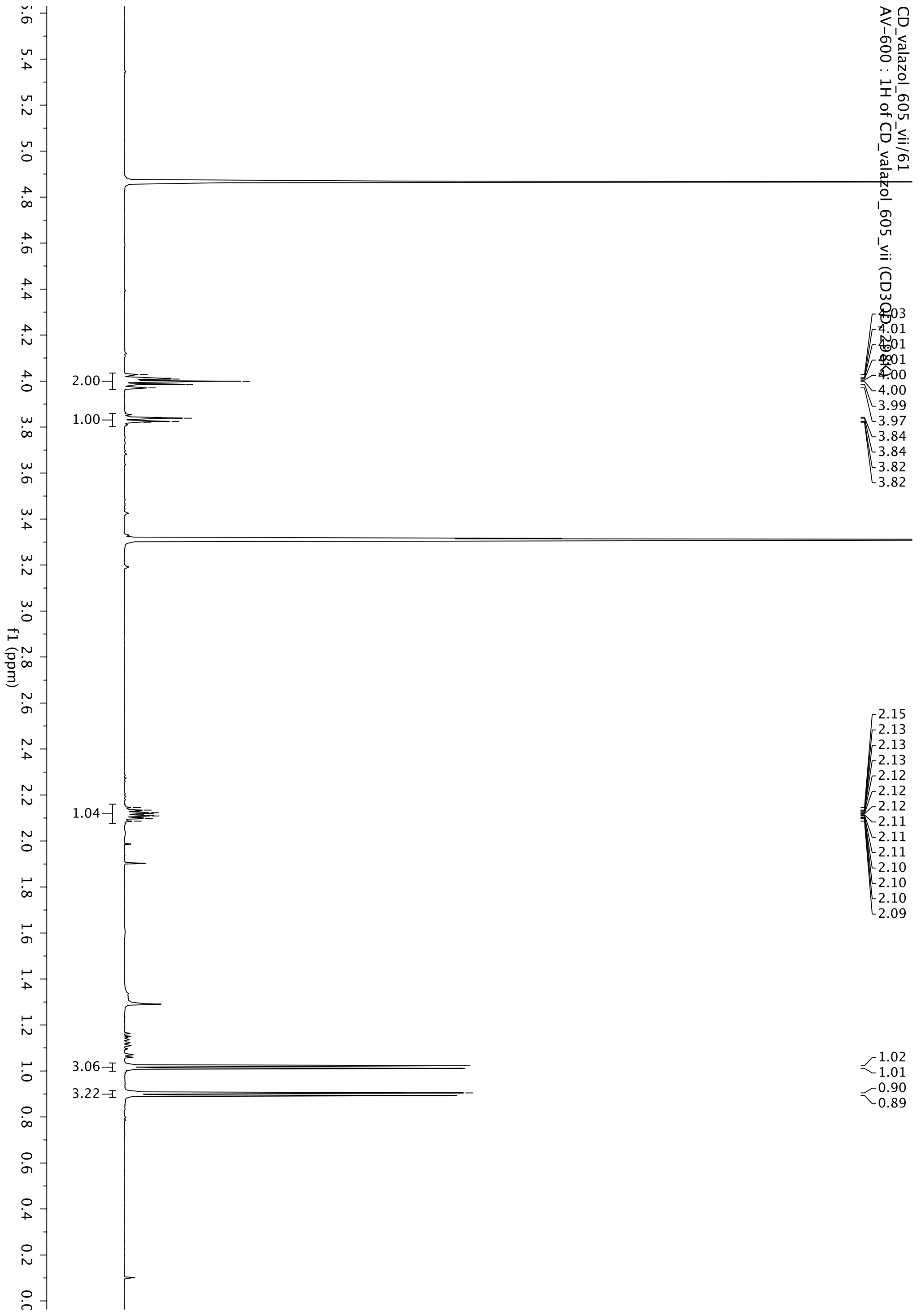

^{1}H-^{1}H COSY NMR Spectrum of isolated valdiazen (**2**) in CD_3OD

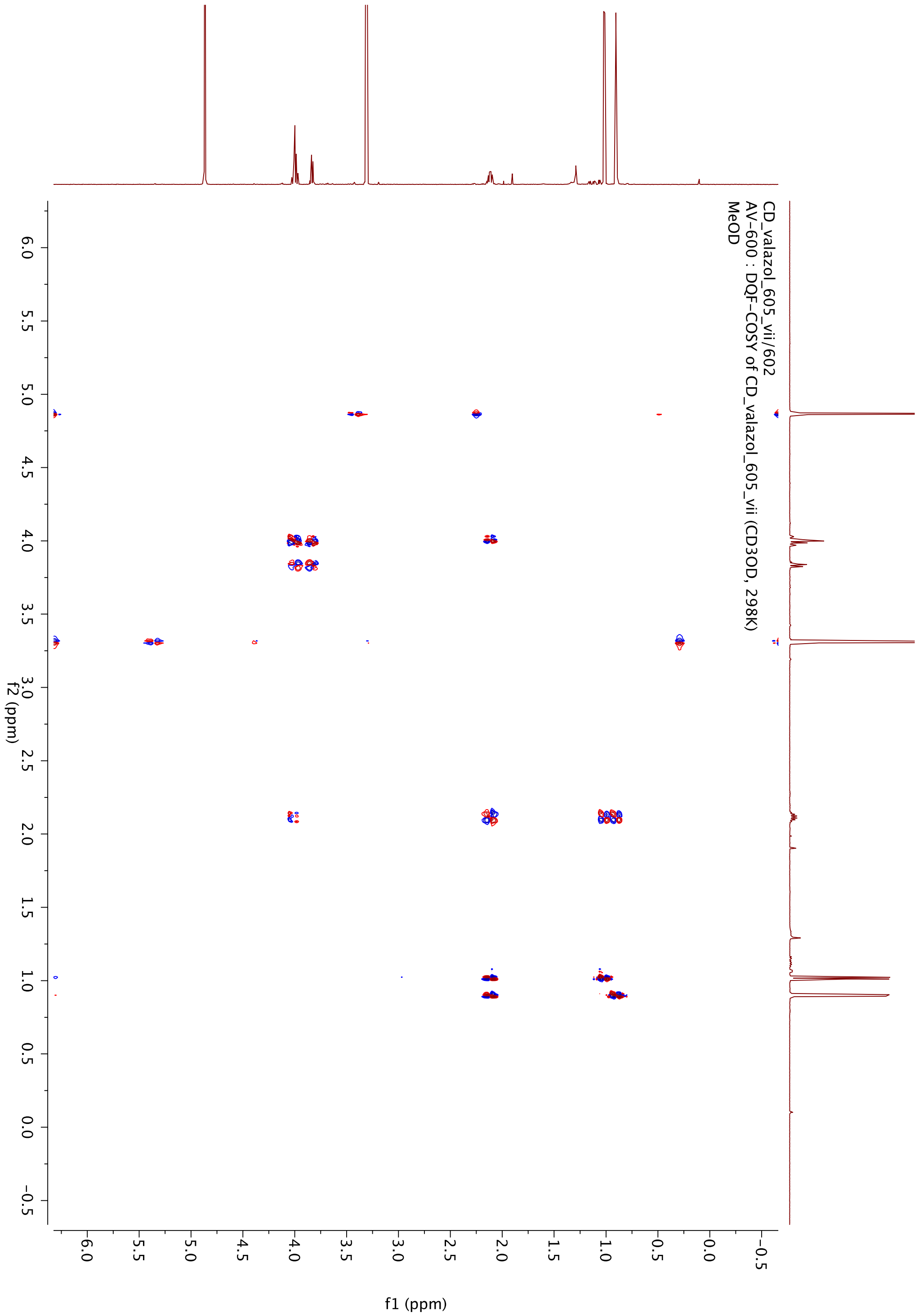

^{1}H-^{13}C HSQC NMR Spectrum of isolated valdiazen (**2**) in CD_3OD

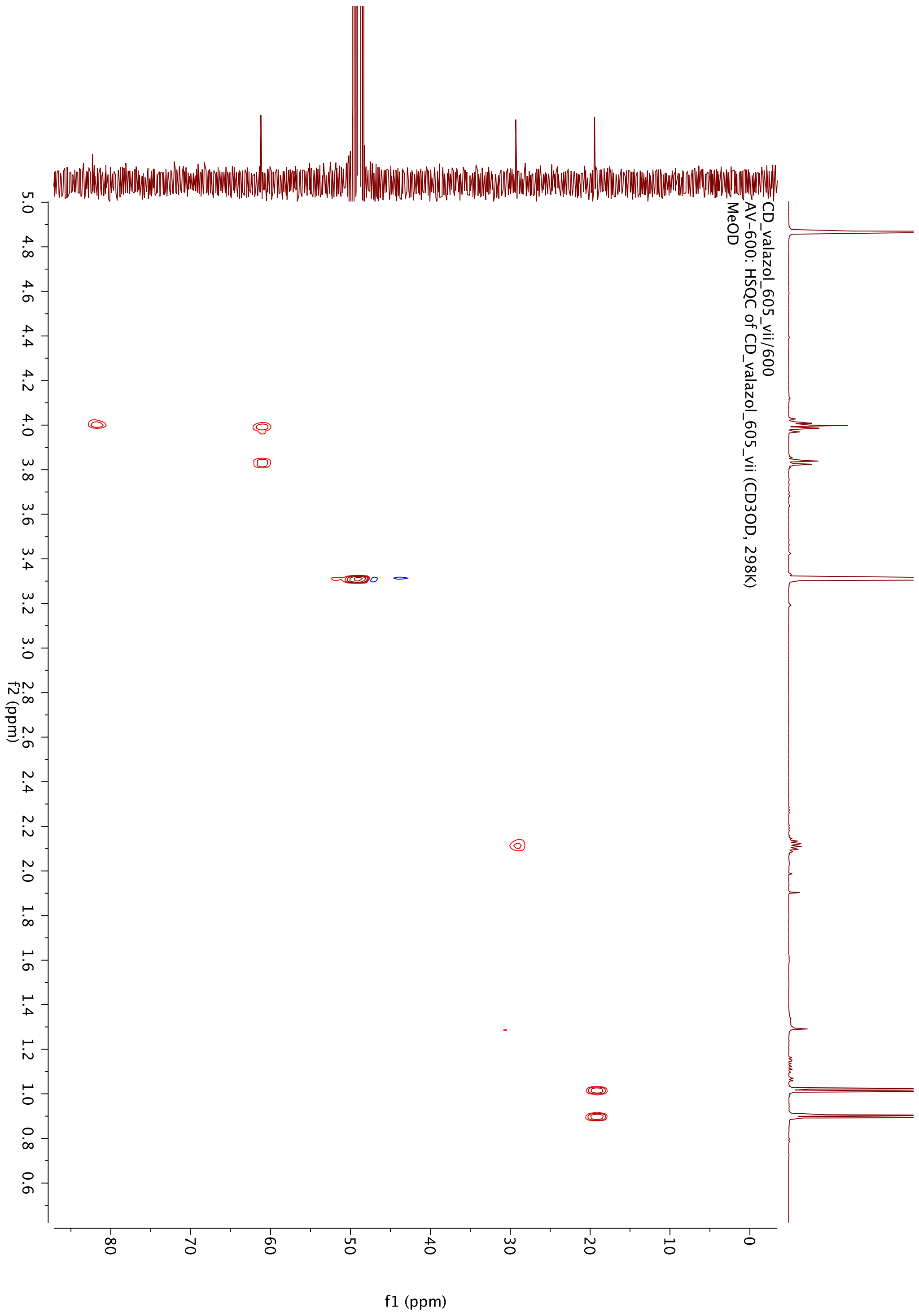

^{1}H-^{13}C HMBC NMR Spectrum of isolated valdiazen (**2**) in CD_3OD

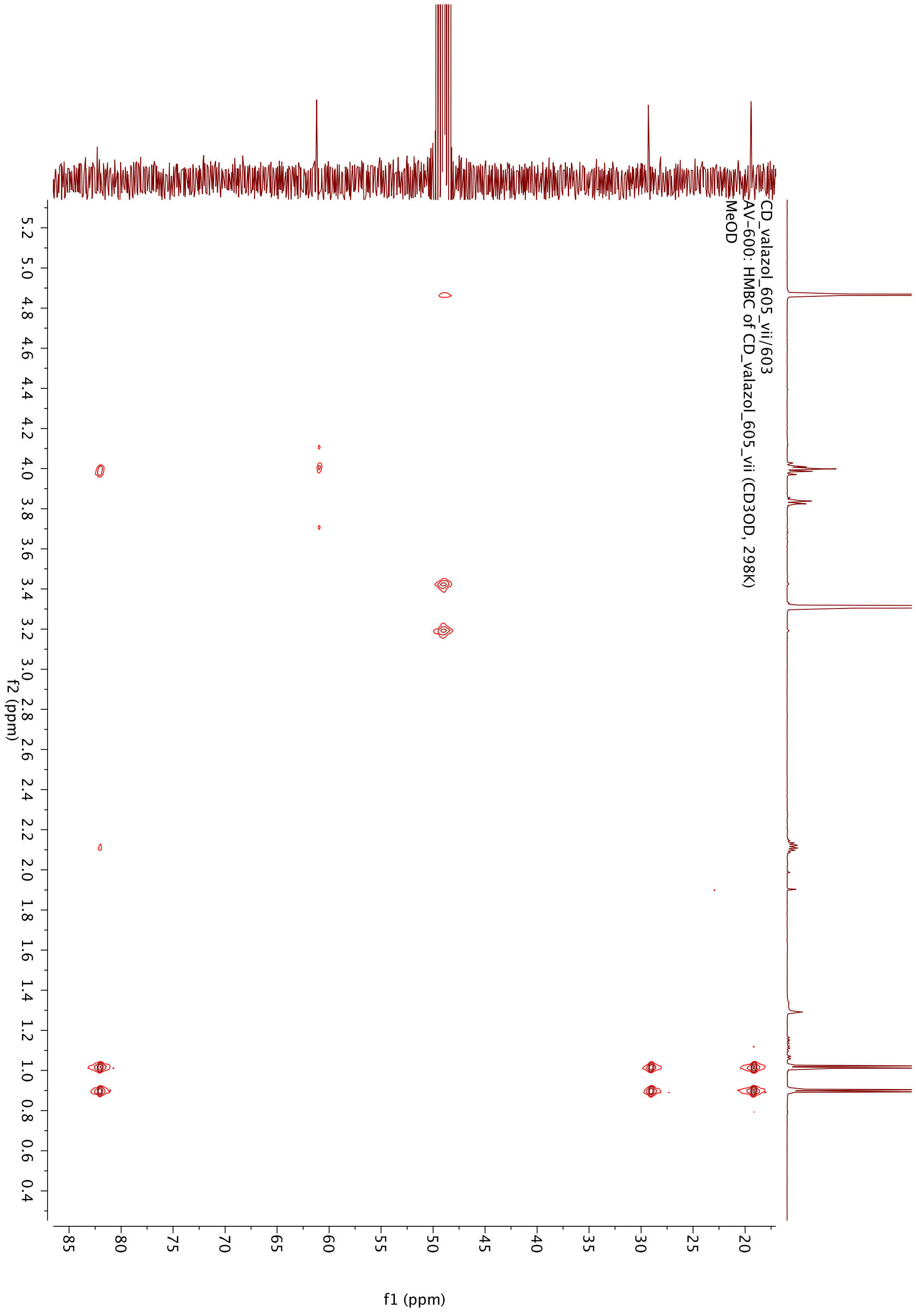

^{13}C-NMR Spectrum of isolated valdiazen (**2**) in CD_3OD

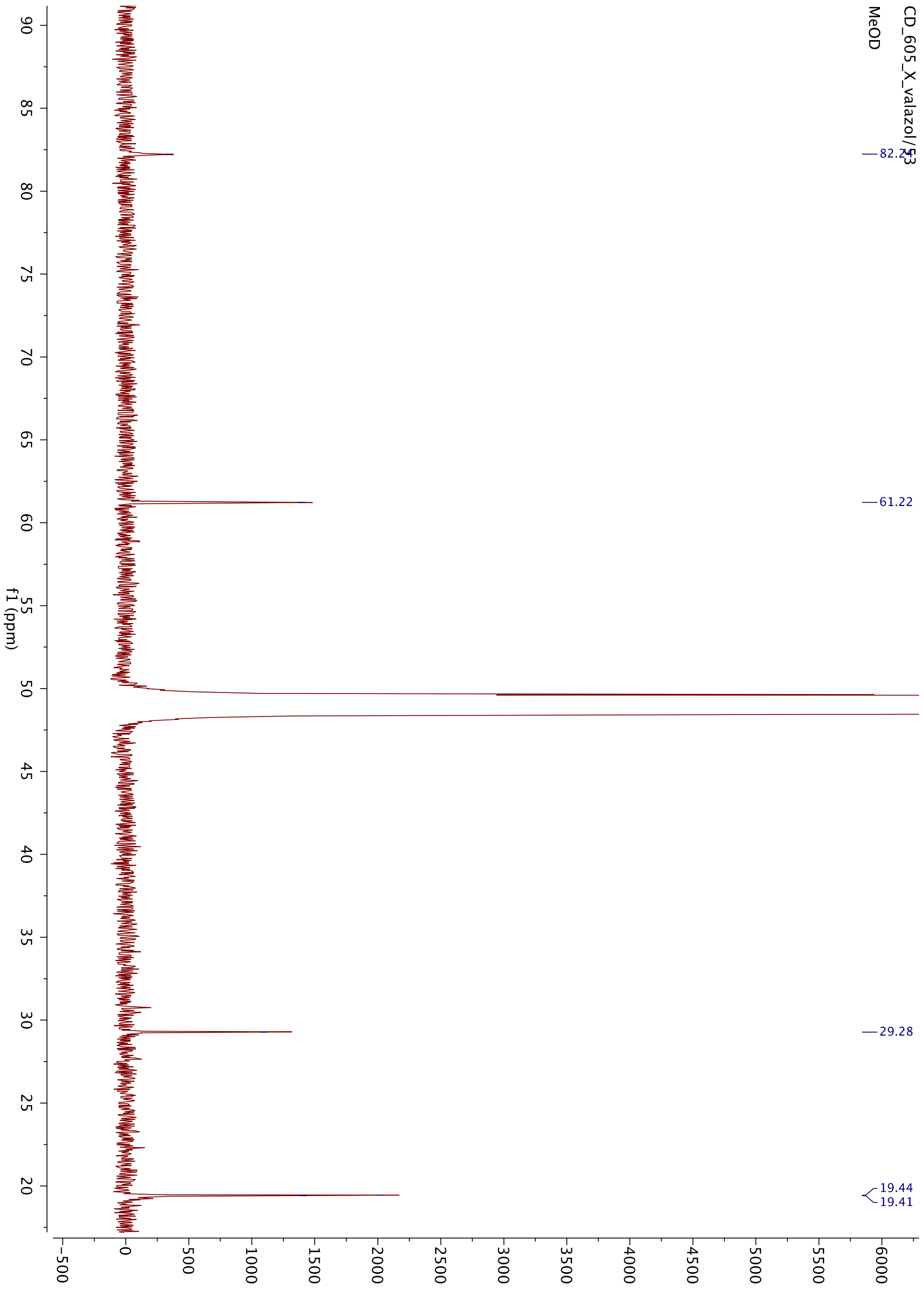

^{1}H-NMR spectra of isolated **2** (top) and synthetic (–)-valdiazen (**12**) (bottom) (600 NMR, CD_3OD)

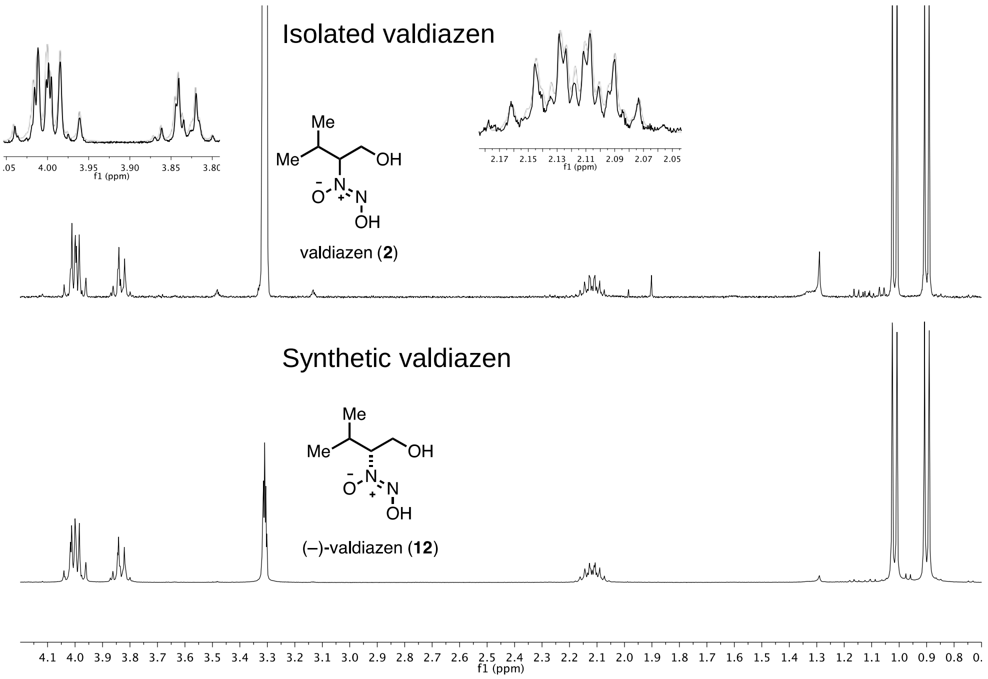

^{13}C-NMR spectra of isolated **2** (top) and synthetic (–)-valdiazen (**12**) (bottom) (500 NMR, CD_3OD)

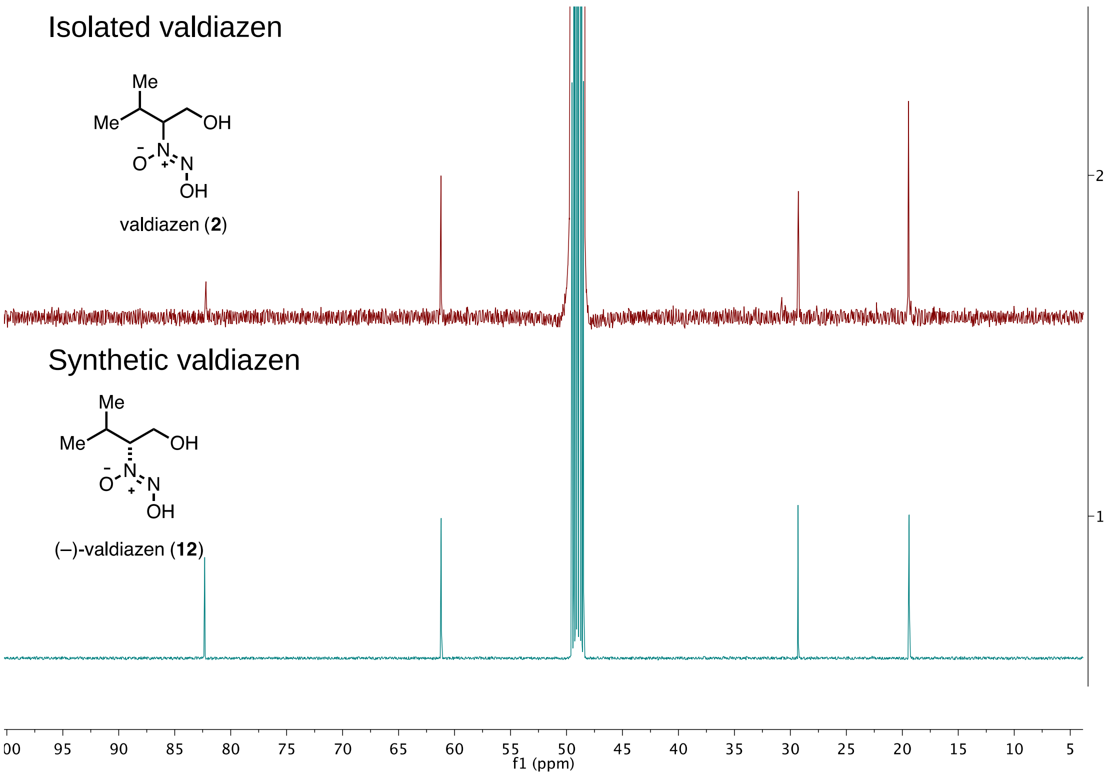

^{1}H-NMR Spectrum of the synthetic (–)-valdiazen (**12**) in CD_3OD

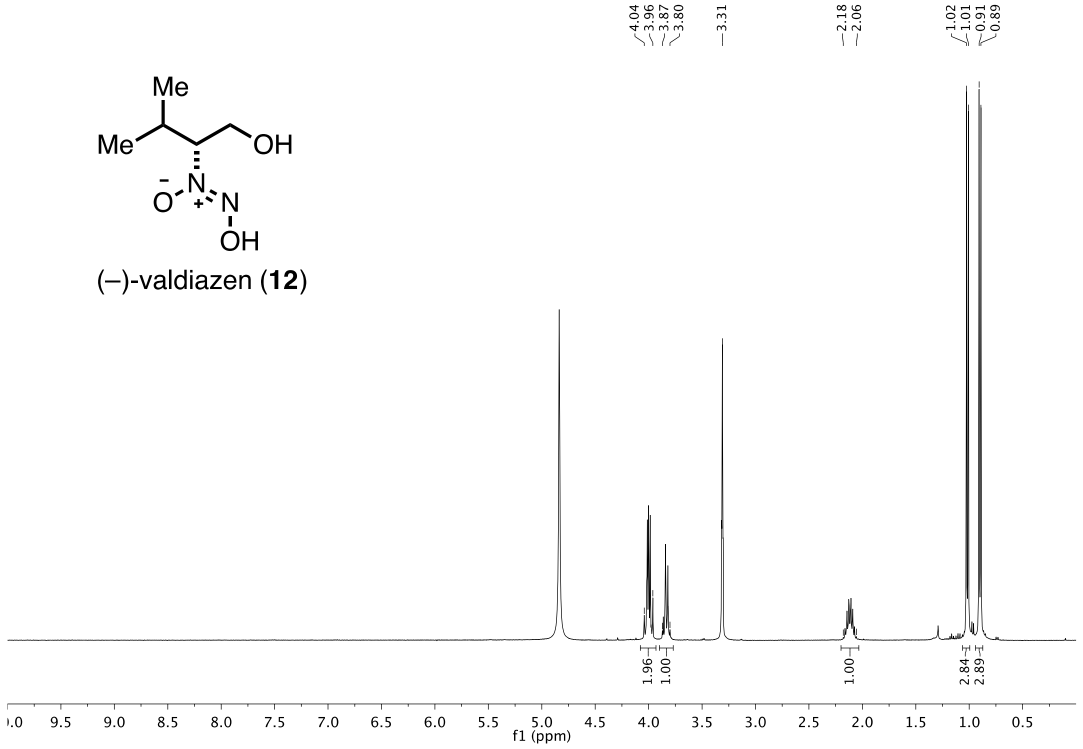

^{13}C-NMR Spectrum of the synthetic (–)-valdiazen (**12**) in CD_3OD

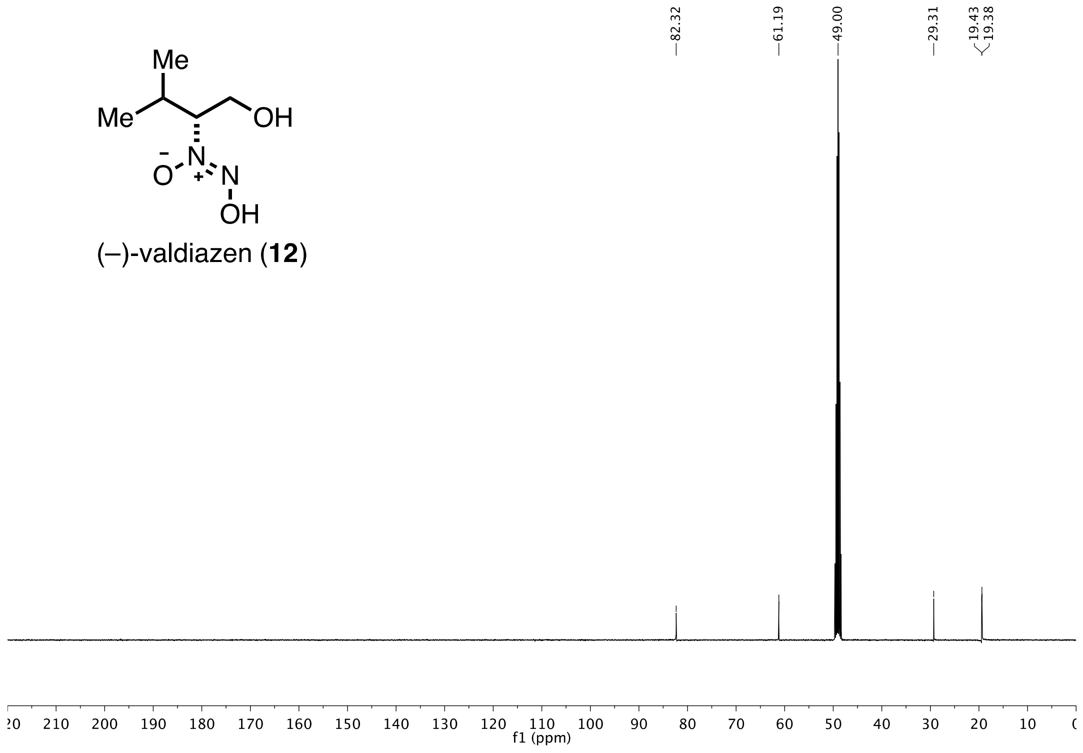

Supplementary Figures

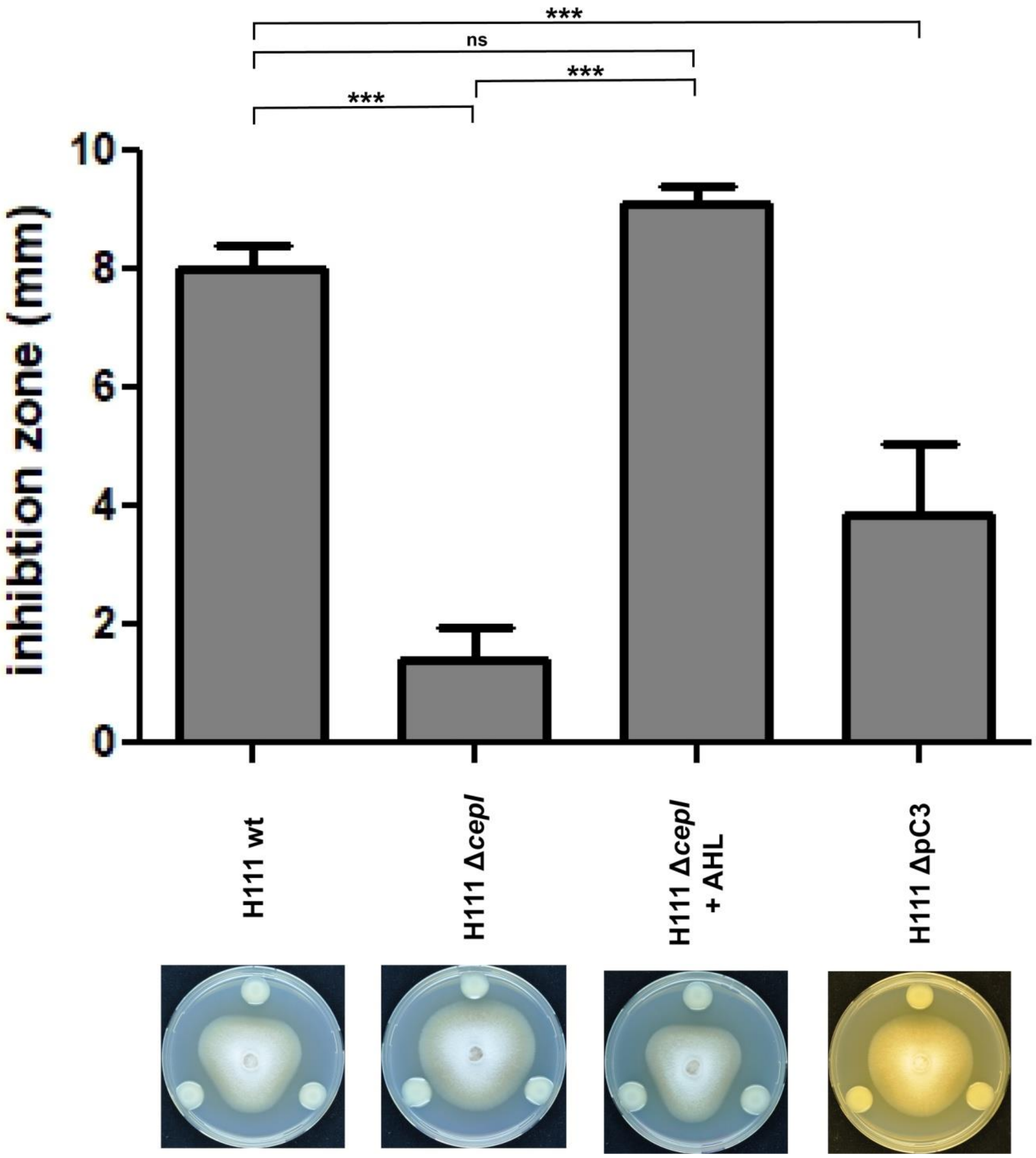

Supplementary Figure 1: Antifungal activity is partially dependent on the CepIR quorum sensing system and the megaplasmid pC3. Reduction of antifungal activity in a Δ*cepI* mutant (H111 Δ*cepI*) and a pC3 null strain (H111 ΔpC3) compared to the wild type strain (H111 wt) against the fungus *F. solani*. External addition of C8-homoserine lactone (200 µM) to the Δ*cepI* mutant (H111 Δ*cepI* + AHL) restores the antifungal phenotype. Results are presented as means and error bars indicate SD. Statistical analysis was performed with One-Way ANOVA and Tukey`s multiple comparison as a post test. Significance is indicated by three stars. $^{***}p<0.001$. n=3. ns = not significant. Representative pictures of the antifungal assays are shown below the respective bars.

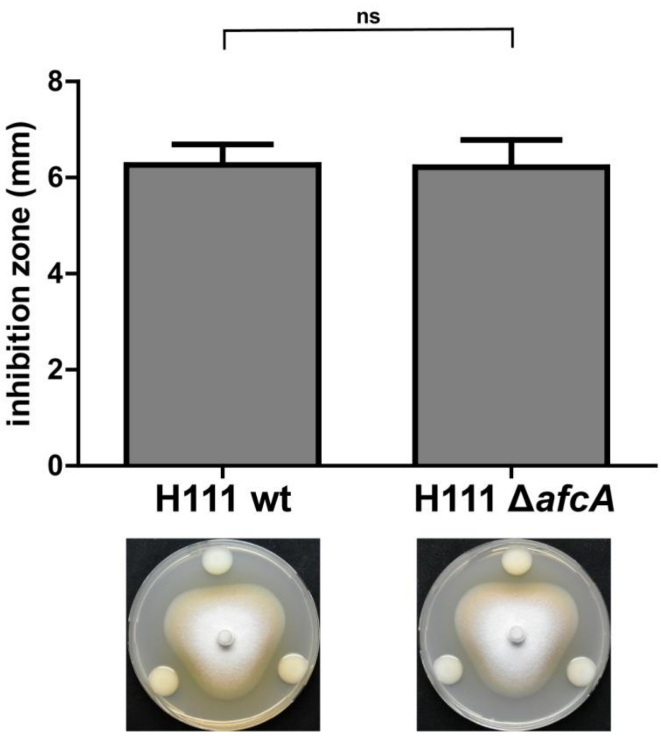

Supplementary Figure 2: AfcA is not involved in antifungal activity of *B. cenocepacia* H111. Antifungal activity against the fungus *F. solani* is not different in an Δ*afcA* mutant (H111 Δ*afcA*) and the wild type strain (H111 wt). Results are represented as means and error bars indicate SD. Statistical analysis was performed with an unpaired two-tailed T-test. p=0.8487; ns = not significant; n=12. Representative pictures of the antifungal assays are shown below the respective bars.

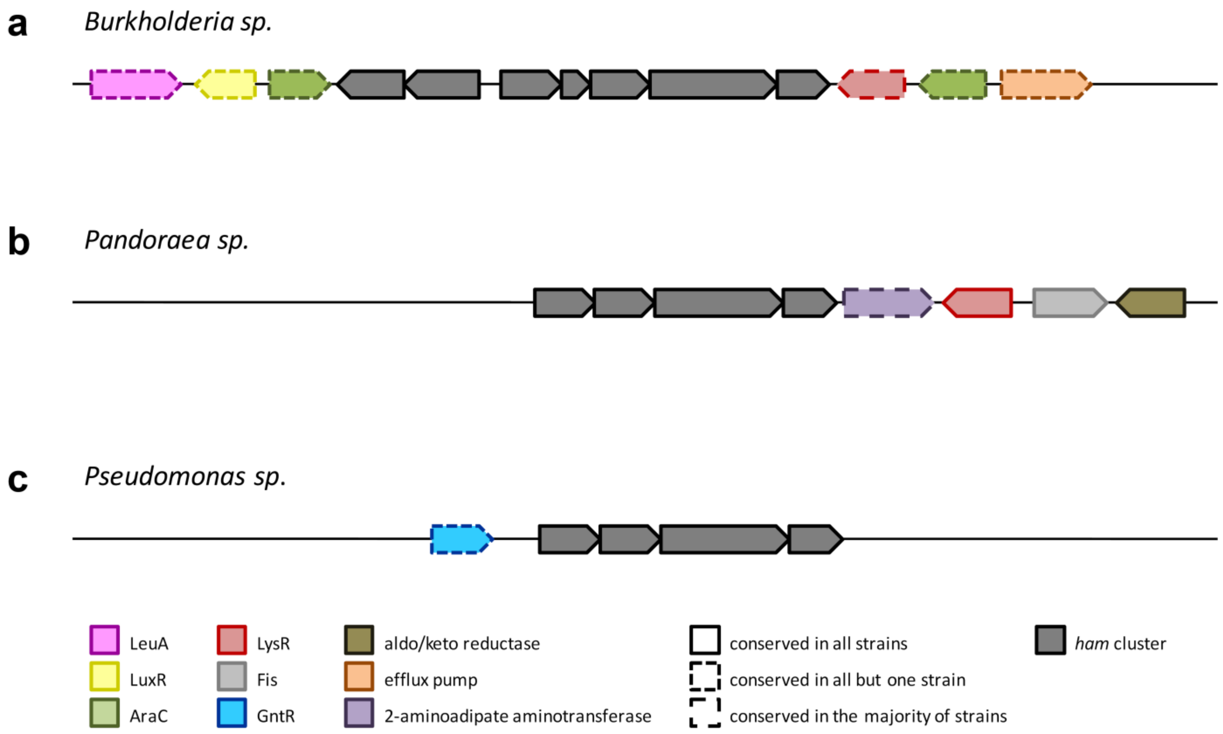

Supplementary Figure 3: Conserved genes immediately upstream and downstream of the *ham* cluster in **(a)** *Burkholderia* strains, **(b)** *Pandoraea* strains and **(c)** *Pseudomonas* strains. Proteins encoded by upstream and downstream genes of the *ham* cluster are color-coded and shown on the bottom of the figure. Full lines represent genes which are present in all strains of the respective genera. Dotted lines represent genes which are present in all except one strain of the respective genus. Broken lines represent genes which are present in the majority of strains from the respective genus. The *ham* cluster and its homologous genes are shown as grey arrows with full black lines.

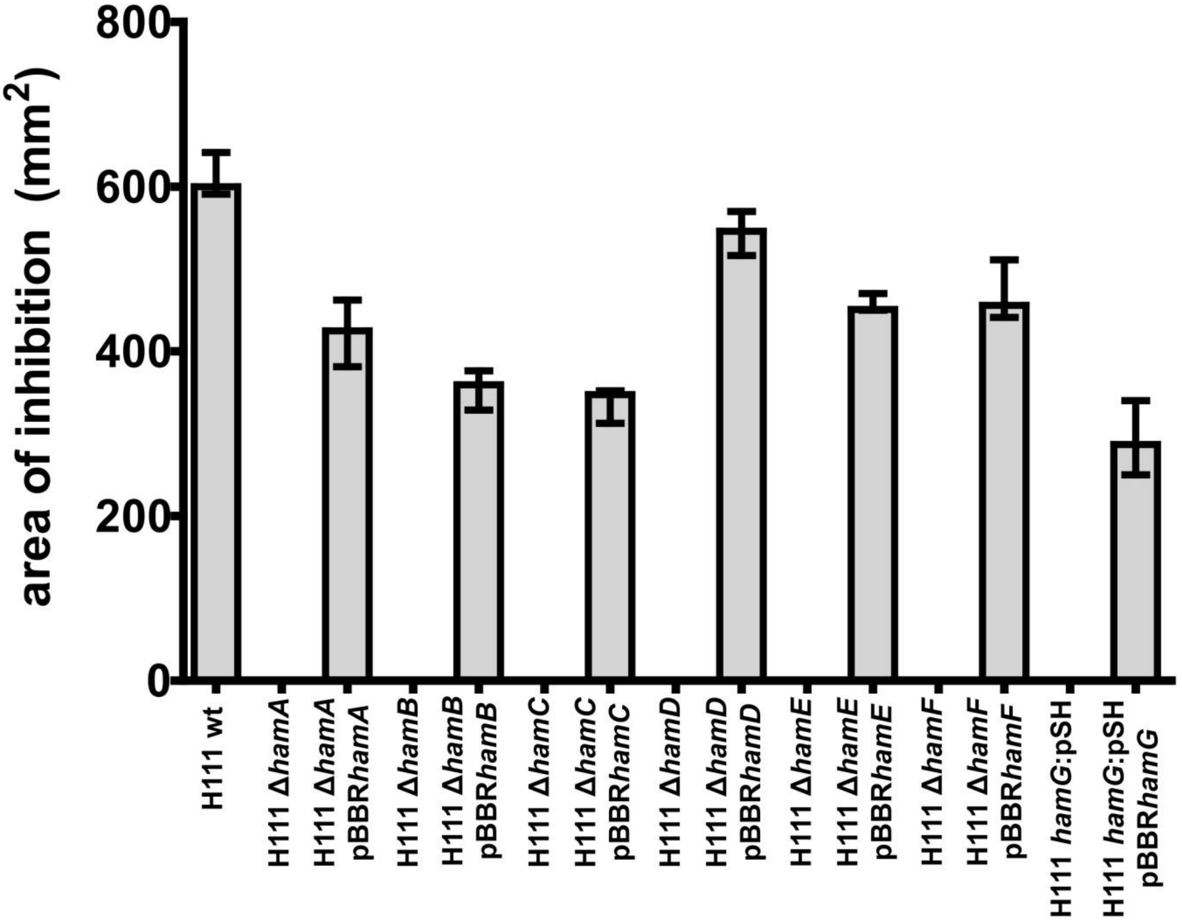

Supplementary Figure 4: All genes of the *ham* cluster are essential for antifungal activity. Antifungal activity of the wild type (H111 wt), the Δ*hamA* mutant (H111 Δ*hamA*), the Δ*hamB* mutant (H111 Δ*hamB*), the Δ*hamC* mutant (H111 Δ*hamC*), the Δ*hamD* mutant (H111 Δ*hamD*), the Δ*hamE* mutant (H111 Δ*hamE*), the Δ*hamF* mutant (H111 Δ*hamF*), the *hamG* mutant (H111 *hamG* :pSH) and the respective *trans* complemented mutants (H111 Δ*hamA* pBBR*hamA*, H111 Δ*hamB* pBBR*hamB*, H111 Δ*hamC* pBBR*hamC*, H111 Δ*hamD* pBBRhamD, H111 Δ*hamE* pBBR*hamE*, H111 Δ*hamF* pBBR*hamF* and H111 *hamG* :pSHpBBR*hamG*). Results are presented as median and error bars indicate interquartile range. n=3.

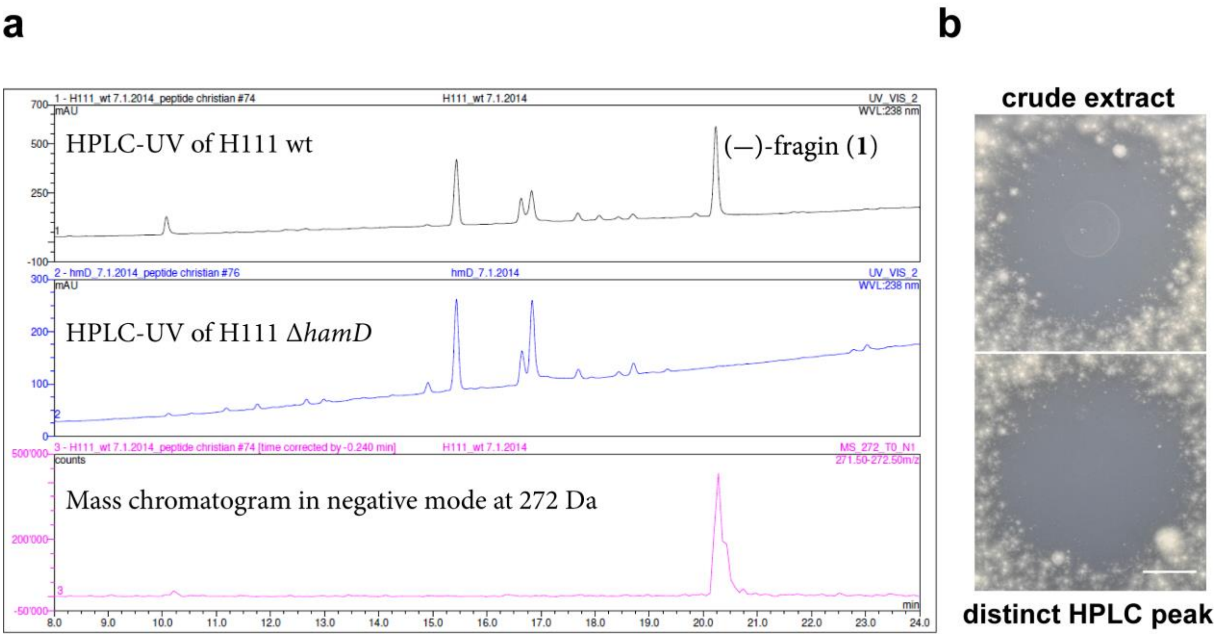

Supplementary Figure 5: (a) Isolation of (–)-fragin (HPLC-MS). **(b)** Bioassay to confirm the antifungal activity of the HPLC-purified compound isolated from the H111 wild type extract (distinct HPLC peak) in comparison with the crude extract. Pictures show the results of an antifungal spray assay using the fungus *F. solani*. Scale bar indicates 10 mm.

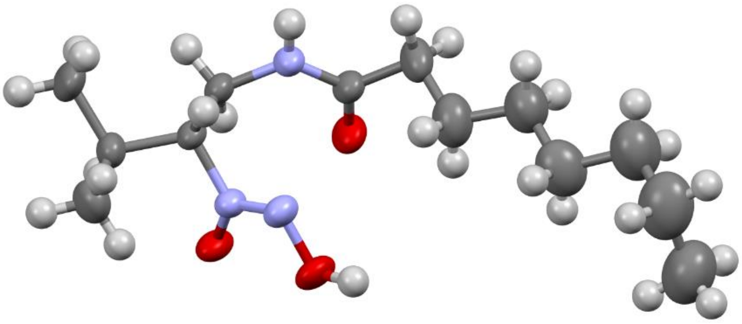

Crystallised from		hexane
Empirical formula		$C_{13}H_{27}N_3O_3$
Formula weight [g mol^{-1}]		273.37
Crystal colour, habit		pale yellow, plate
Crystal dimensions [mm]		$0.12 \times 0.54 \times 0.75$
Temperature [K]		230(1)
Crystal system		orthorhombic
Space group		$P2_12_12_1$ (#19)
Z		4
Reflections for cell determination		7912
2θ range for cell determination [°]		9–146
Unit cell parameters	a [Å]	5.78770(8)
	b [Å]	9.54793(12)
	c [Å]	29.2360(3)
	α, β, γ [°]	90, 90, 90
	V [Å^3]	1615.60(3)
$F(000)$		600
D_X [g cm^{-3}]		1.124
μ(Cu $K\alpha$) [mm^{-1}]		0.648
Scan type		ω
2θ(max) [°]		145.8
Transmission factors (min; max)		0.415; 1.000
Total reflections measured		11534
Symmetry independent reflections		3143
R_{int}		0.032
Reflections with $I > 2\sigma(I)$		2986
Reflections used in refinement		3143
Parameters refined; restraints		183; 138
Final	$R(F)$ [$I > 2\sigma(I)$ reflections]	0.0628
	$wR(F^2)$ (all data)	0.1985
Weights:		$w = [\sigma^2(F_o^2) + (0.1201P)^2 + 0.5685P]^{-1}$ where $P = (F_o^2 + 2F_c^2)$
Goodness of fit		1.075
Absolute structure parameter		-0.04(9)
Final Δ_{max}/σ		0.000
$\Delta\rho$ (max; min) [e Å^{-3}]		0.45; -0.38
$\sigma(d_{(C–C)})$ [Å]		0.004 – 0.006

Supplementary Figure 6: Crystallographic data for the isolated (−)-(*R*)-fragin (**1**)

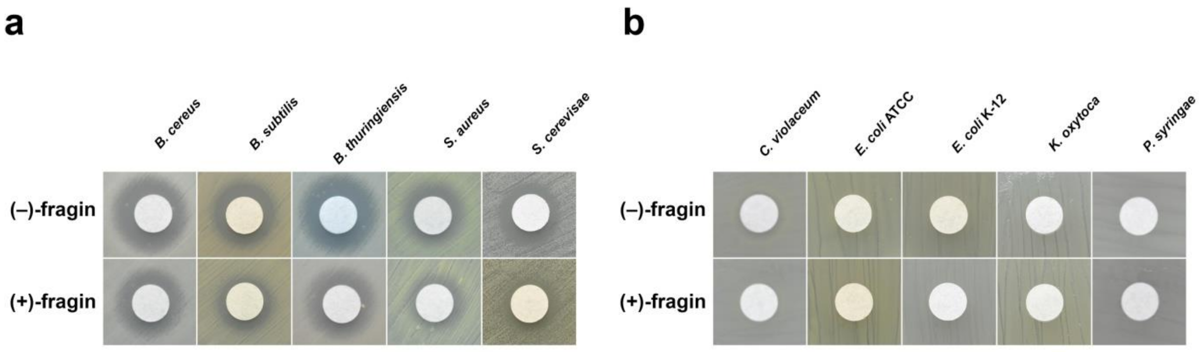

c

	MIC range (µg/ml)				
Compound	*Bacillus cereus*	*Staphylococcus aureus*	*Pseudomonas putida*	*Escherichia coli*	*Klebsiella oxytoca*
(–)-fragin	16	32	ND	128	128-ND
(+)-fragin	16-64	32-64	ND	128	128-ND

Supplementary Figure 7: Antibacterial activity of fragin. **(a)** Disc diffusion assays of 20 µg synthetic (–)- and (+)-fragin to determine activity against the Gram-positive bacteria *Bacillus cereus*, *Bacillus subtilis*, *Bacillus thuringiensis*, *Staphylococcus aureus* and the yeast *Saccharomyces cerevisiae*. Representative pictures are shown. **(b)** Disc diffusion assays of 20 µg synthetic (–)- and (+)-fragin to determine activity against the Gram-negative bacteria *Chromobacterium violaceum*, *Eschericia coli ATCC*, *E. coli K-12*, *Klebsiella oxytoca* and *Pseudomonas syringae*. Representative pictures are shown. **(c)** Minimal inhibitory concentration (MIC) range of synthetic (–)- and (+)-fragin against a panel of selected Gram-positive and Gram-negative bacteria. MIC tests were performed in technical triplicates from biological triplicates. ND: not determined, bacteria were not inhibited by the highest tested concentration (128 µg/ml).

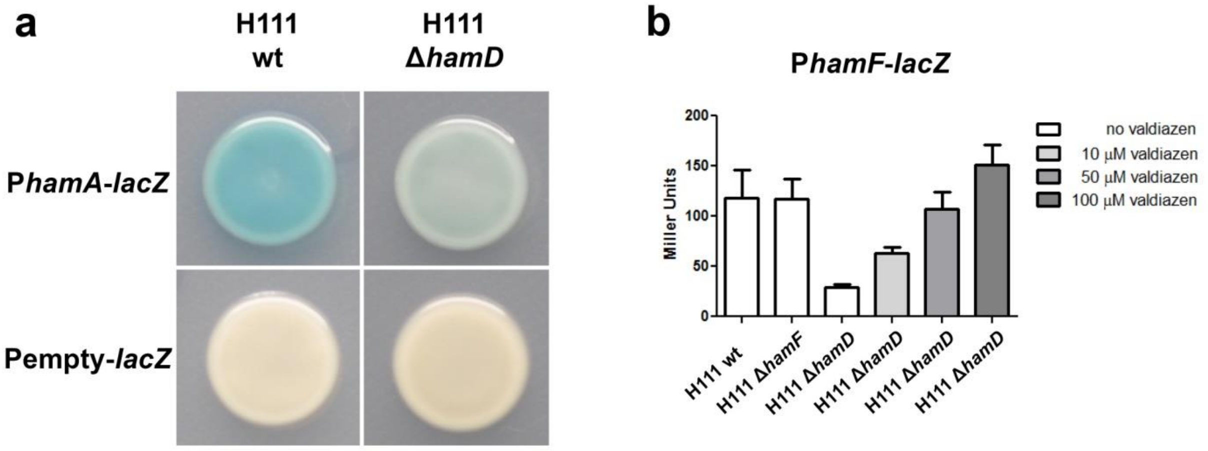

Supplementary Figure 8: The *ham* gene cluster regulates its own biosynthesis by a positive feedback loop. **(a)** Representative pictures of the promoter activity of the *hamABCDE* operon (P*hamA*-*lacZ*) in the wildt ype (H111 wt) and a Δ*hamD* mutant (H111 Δ*hamD*) background observed on agar plates supplemented with 40 µg/ml X-gal as substrate. The intensity of the blue coloration of the H111 wild type colony indicates a higher promoter activity than in the H111 Δ*hamD* background. Empty vector controls with pSU11 (empty vector) showed no detectable promoter activity in either strain background. **(b)** The promoter activity of the *hamFG* operon (P*hamF*-*lacZ*) was comparable in the wild type strain (H111 wt) and the *hamF* mutant strain (H111 Δ*hamF*). The *hamD* mutant strain (H111 Δ*hamD*) showed only very low promoter activity which could be activated by the addtion of synthetic valdiazen. Results are presented as means and error bars represent SD. n=3.

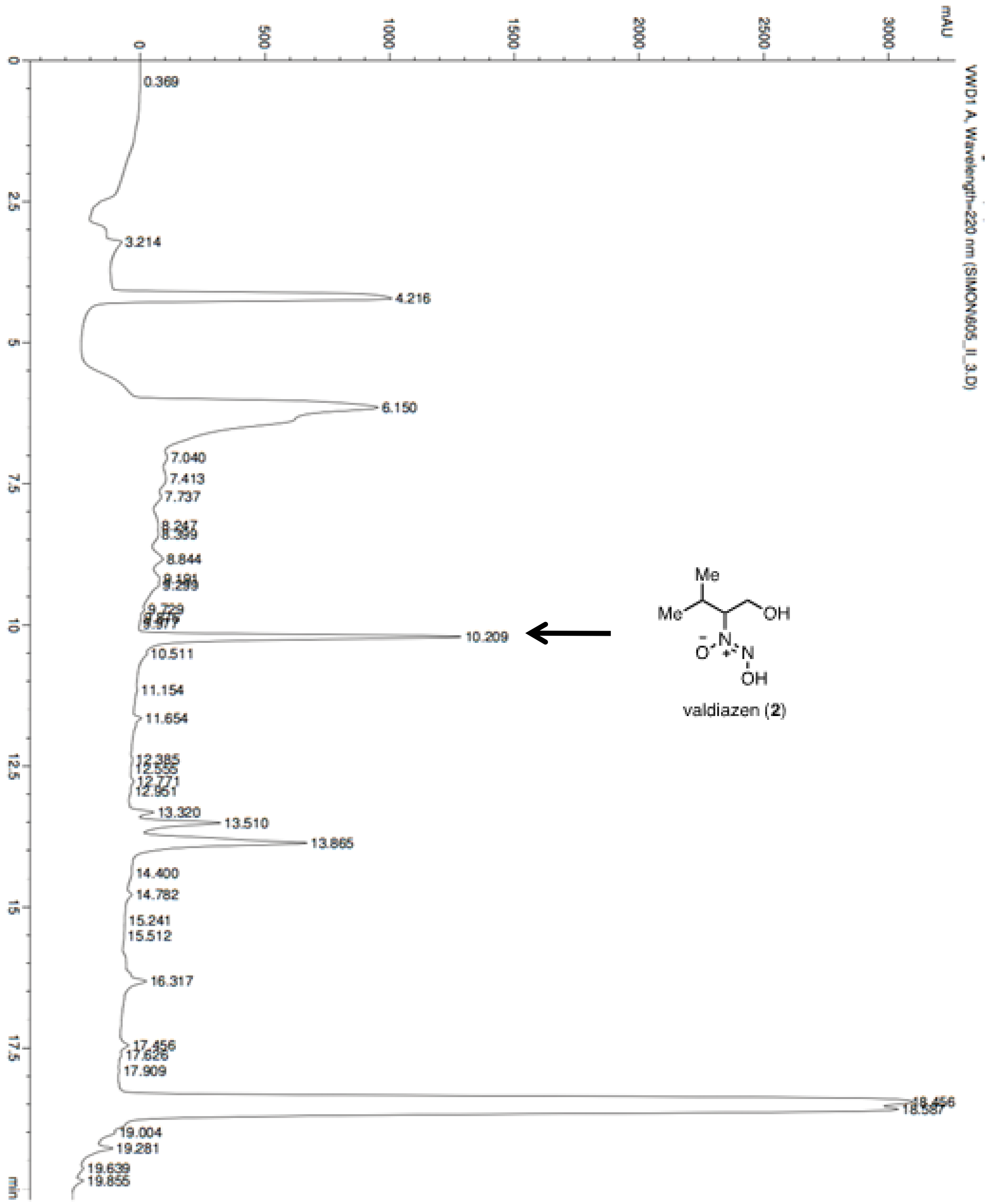

Supplementary Figure 9: HPLC chromatogram of the H111 Δ*hamF* extract after acid base extraction. Valdiazen (**2**) is eluting with a retention time of 10.2 minutes.

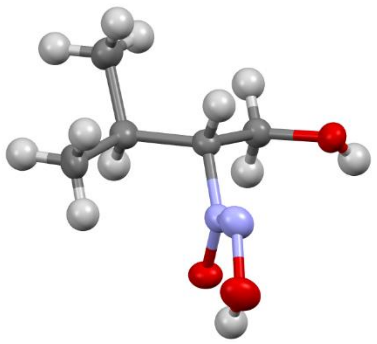

Crystallised from		from the pure oil
Empirical formula		$C_5H_{12}N_2O_3$
Formula weight [g mol^{-1}]		148.17
Crystal colour, habit		colourless, plate
Crystal dimensions [mm]		0.03 × 0.22 × 0.22
Temperature [K]		160(1)
Crystal system		orthorhombic
Space group		$P2_12_12_1$ (#19)
Z		4
Reflections for cell determination		3586
2θ range for cell determination [°]		11–147
Unit cell parameters	a [Å]	6.14476(18)
	b [Å]	9.3078(3)
	c [Å]	13.4817(5)
	α, β, γ [°]	90, 90, 90
	V [Å^3]	771.08(5)
$F(000)$		320
D_x [g cm^{-3}]		1.276
μ(Cu $K\alpha$) [mm^{-1}]		0.890
Scan type		ω
$2\theta_{(max)}$ [°]		148.4
Transmission factors (min; max)		0.623; 1.000
Total reflections measured		7248
Symmetry independent reflections		1558
R_{int}		0.072
Reflections with $I > 2\sigma(I)$		1437
Reflections used in refinement		1558
Parameters refined		101
Final	$R(F)$ [$I > 2\sigma(I)$ reflections]	0.0379
	$wR(F^2)$ (all data)	0.0999
Weights:		$w = [\sigma^2(F_o^2) + (0.0633P)^2]^{-1}$ where $P = (F_o^2 + 2F_c^2)/3$
Goodness of fit		1.042
Absolute structure parameter		0.1(2)
Final Δ_{max}/σ		0.000
$\Delta\rho$ (max; min) [e Å^{-3}]		0.19; -0.14
$\sigma(d_{(C-C)})$ [Å]		0.003

Supplementary Figure 10: Crystallographic data for the synthetic (−)-(*R*)-valdiazen (**12**).

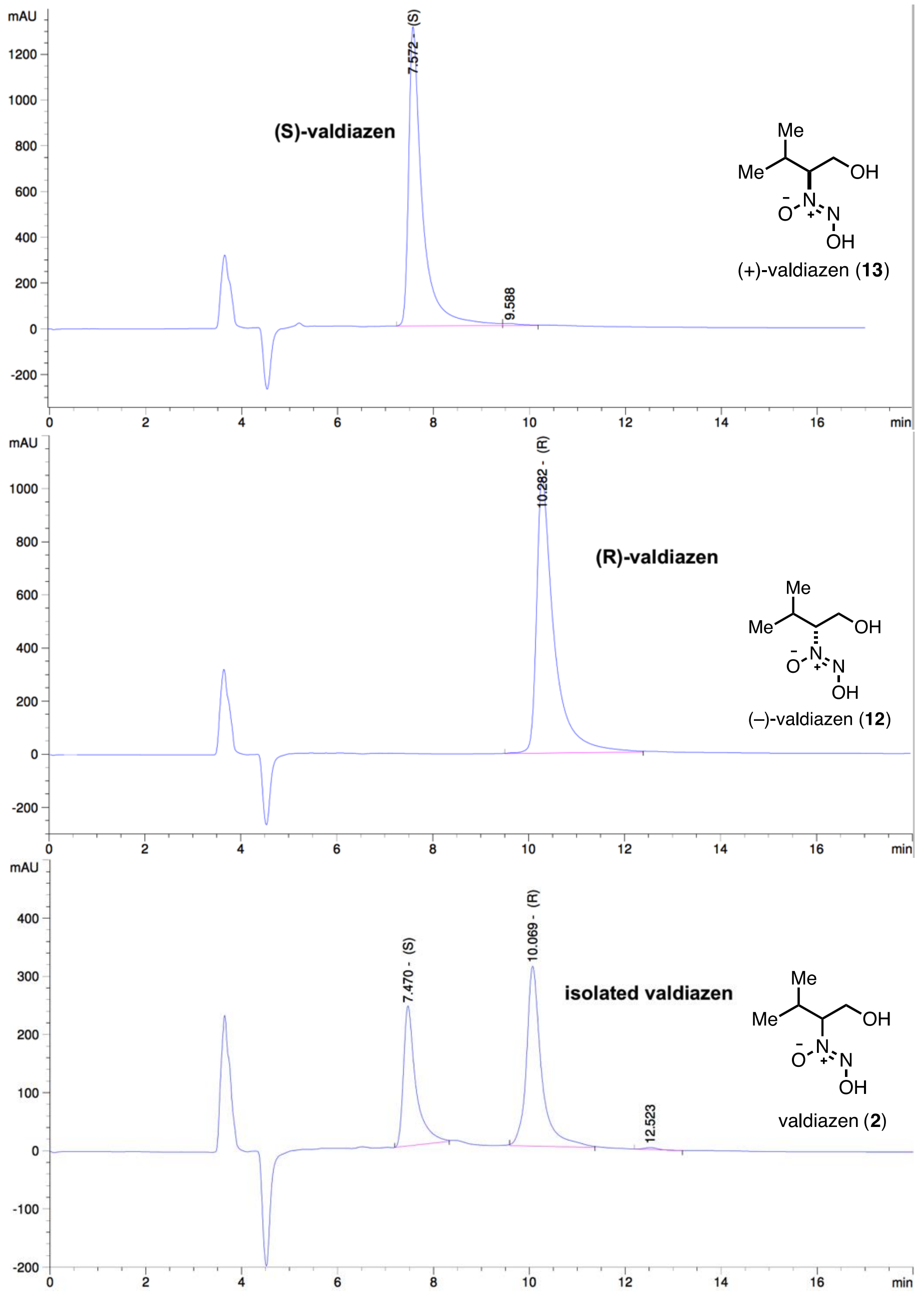

Supplementary Figure 11: Chiral HPLC analysis of synthetic (+)-valdiazen (**13)** and (–)-valdiazen (**12**) and from *B. cenocepacia* H111 isolated valdiazen (**2**).

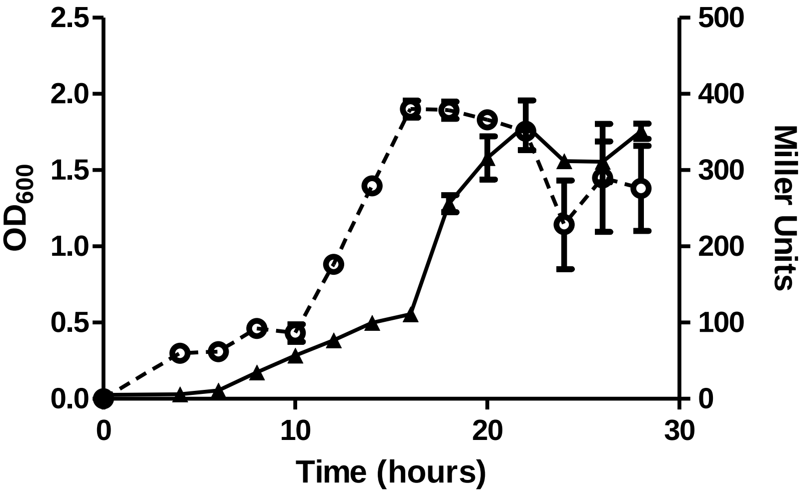

Supplementary Figure 12: Promoter activity of the *hamABCDE* operon is induced at the late exponential growth phase. Bacterial growth (OD_{600}) and activity of the *ham* promoter (Miller Units) measured over a time period of 28 hours. Triangles connected by a full line represent measurements of bacterial growth at OD_{600} (left Y axis). Empty circles connected by a dashed line represent measurements of promoter activity in Miller Units (right Y axis). Results are represented as mean and error bars indicate SD. n=3.

a

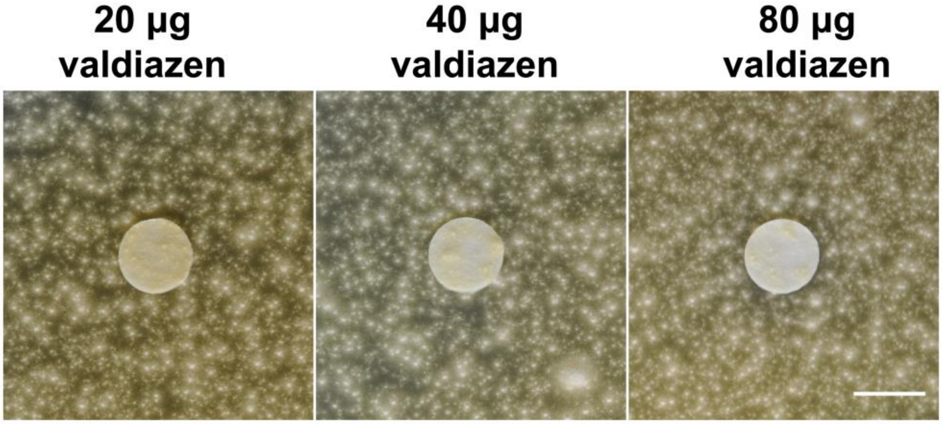

b

	MIC range (µg/ml)				
Bacterium	*Bacillus cereus*	*Staphylococcus aureus*	*Pseudomonas putida*	*Escherichia coli*	*Klebsiella oxytoca*
valdiazen	ND	ND	ND	128	128-ND

Supplementary Figure 13: Valdiazen (**2**) does not exhibit antibiotic activity. **(a)** Disc diffusion assays of synthetic (–)-valdiazen to determine its antifungal activity. Representative pictures of an antifungal spray assay against the fungus *F. solani* are shown. Scale bar indicates 10 mm. **(b)** MIC range of synthetic (–)-valdiazen against a panel of selected Gram-positive and Gram-negative bacteria. MIC tests were performed in technical triplicates from biological triplicates. ND: not determined, bacteria were not inhibited by the highest tested concentration (128 µg/ml).

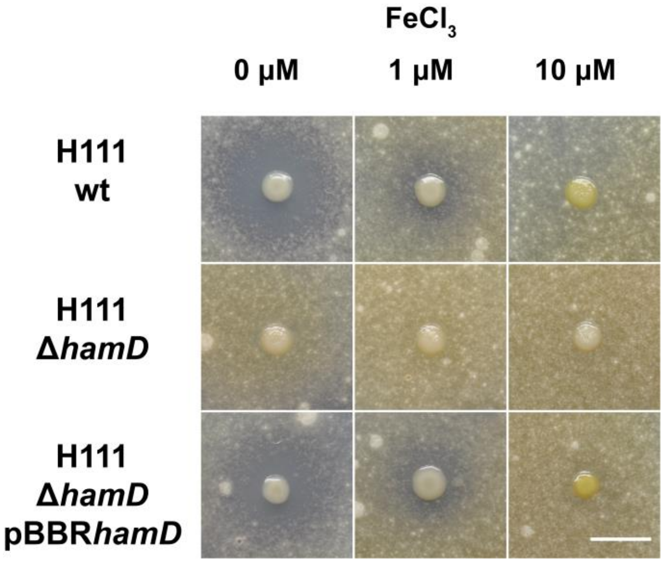

Supplementary Figure 14: Antifungal activity is lost in the presence of excess amounts of iron. Addition of 1 µM $FeCl_3$ to standard MEA plates diminishes antifungal activity and addition of 10 µM $FeCl_3$ completely abrogates antifungal activity in the wild type strain (H111 wt) and the complemented Δ*hamD* mutant (H111 Δ*hamD* pBBR*hamD*). No antifungal acitivity is observed for the Δ*hamD* mutant strain H111 Δ*hamD* and this phenotype is not changed by the addition of $FeCl_3$. Representative pictures of antifungal spray assays against the fungus *F. solani* are shown. Scale bar indicates 10 mm.

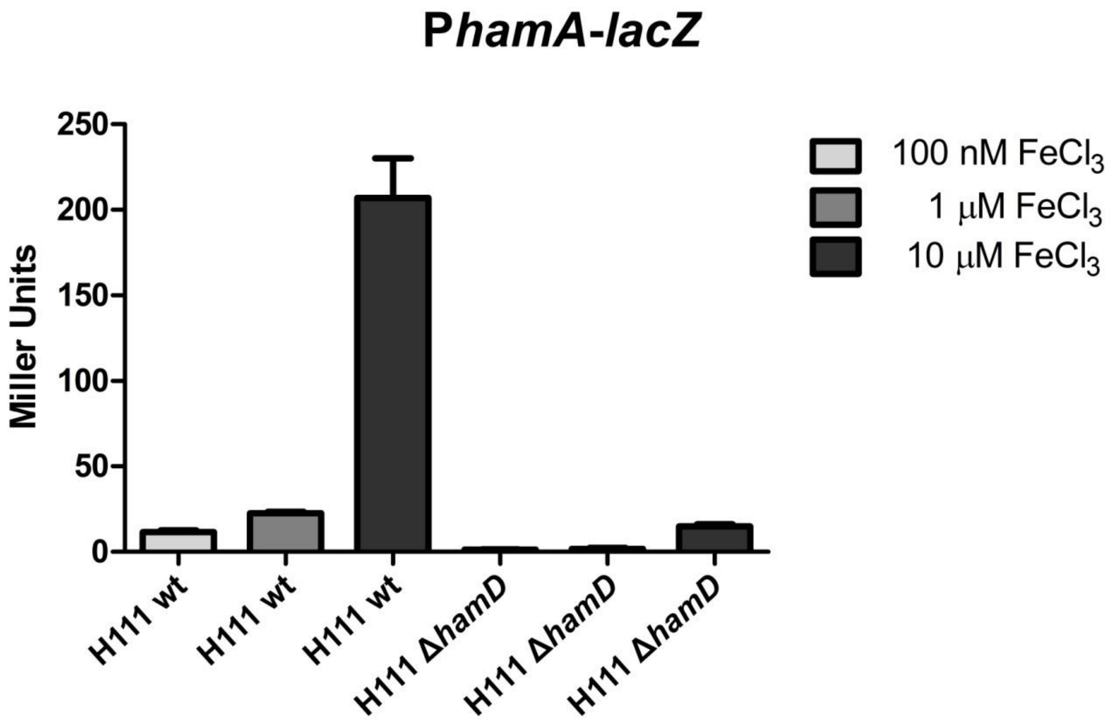

Supplementary Figure 15: Iron positively regulates the promoter of the *hamABCDE* operon. Activity of the *ham* promoter (P*hamA-lacZ*) in the wild type (H111 wt) and the Δ*hamD* mutant (H111 Δ*hamD*) background in the presence of different concentrations of iron ($FeCl_3$). The *hamA* promoter is activated in the presence of large amounts of iron. Results are presented as means and error bars indicate SD. n=3

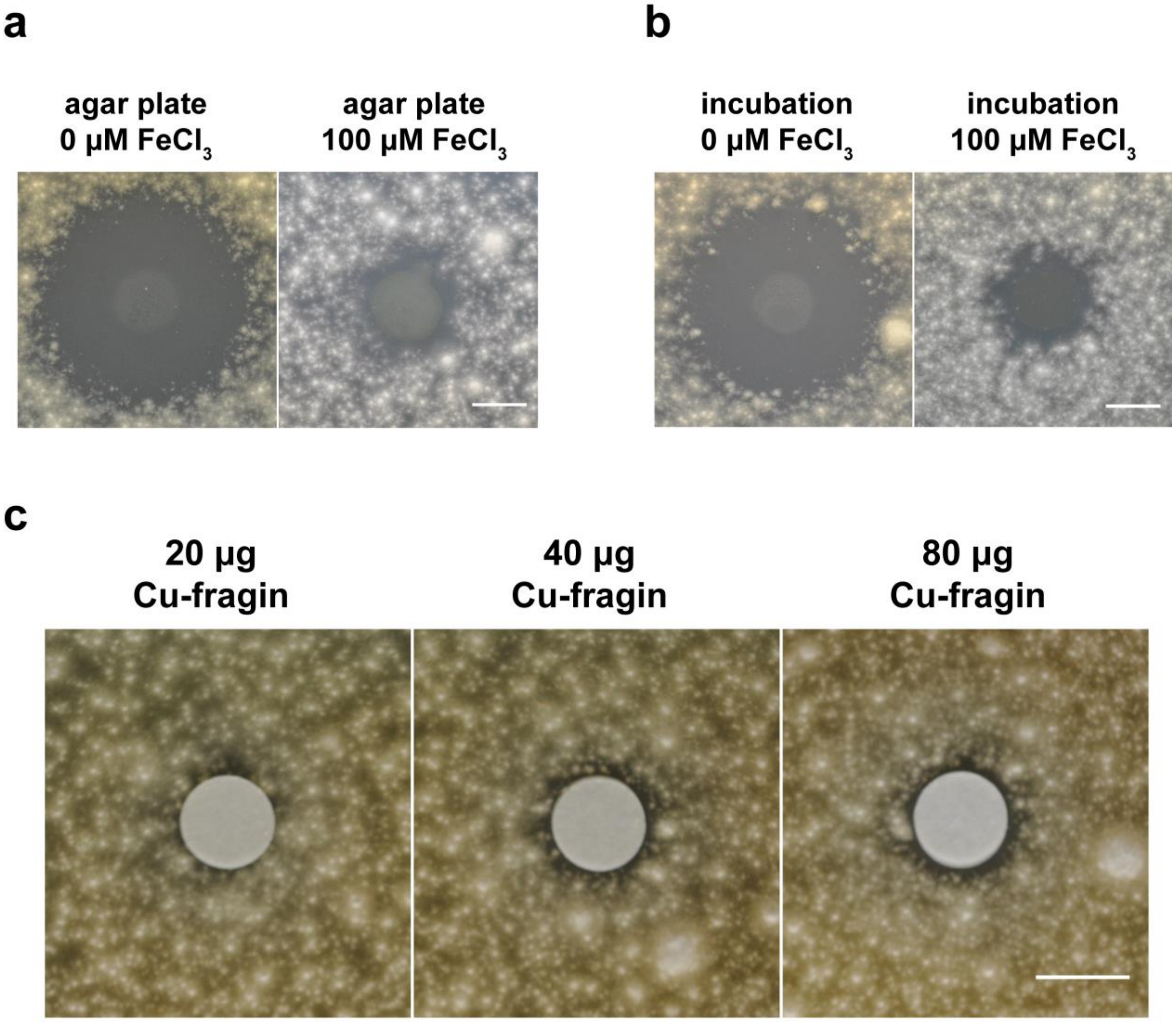

d

	MIC range (µg/ml)				
Bacterium	*Bacillus cereus*	*Staphylococcus aureus*	*Pseudomonas putida*	*Escherichia coli*	*Klebsiella oxytoca*
Cu-fragin	32-64	64	ND	ND	128-ND

Supplementary Figure 16: Metals reduce the antibiotic activity of fragin. **(a)** Crude fragin extracts from H111 wild type cultures show highly reduced antifungal activity on **(a)** agar plates containing iron (agar plate 100 µM $FeCl_3$) or **(b)** when pre-incubated with iron overnight (incubation 100 µM $FeCl_3$).. Representative pictures of antifungal spray assays against the fungus *F. solani* are shown. Scale bar indicates 10 mm. **(c)** Disc diffusion assays showing the loss of antifungal activity of synthetic Cu-fragin complex. Representative pictures of antifungal spray assays against the fungus *F. solani* are shown. Scale bar represents 10 mm. **(d)** MIC range of a synthetic Cu-fragin complex against a panel of selected Gram-positive and Gram-negative bacteria shows that antibacterial activity against Gram-positive bacteria is not lost by copper complexation. MIC tests were performed in technical triplicates from biological triplicates. ND: not determined, bacteria were not inhibited by the highest tested concentration (128 µg/ml).

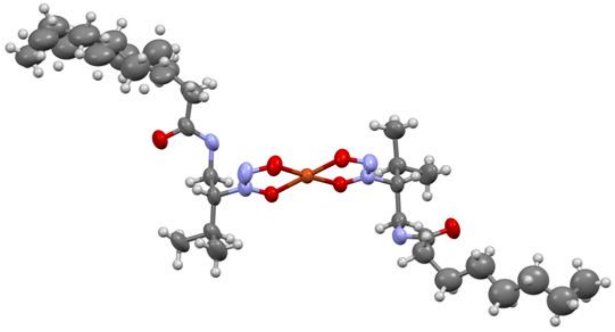

Empirical formula		$C_{26}H_{50}Cu_1N_6O_6$
Formula weight [g mol^{-1}]		606.27
Crystal colour, habit		blue, needle
Crystal dimensions [mm]		0.03 × 0.04 × 0.19
Temperature [K]		123(1)
Crystal system		trigonal
Space group		$P3_221$
Z		3
Reflections for cell determination		9921
2θ range for cell determination [°]		8–136
Unit cell parameters	a [Å]	14.3908(18)
	b [Å]	14.3908(18)
	c [Å]	13.6432(18)
	α, β, γ [°]	90, 90, 120
	V [Å^3]	2446.7(5)
$F(000)$		975
D_x [g cm^{-3}]		1.234
μ(Mo $K\alpha$) [mm^{-1}]		1.312
Scan type		ϕ and ω
$2\theta_{(max)}$ [°]		137.3
Transmission factors (min; max)		0.95; 0.96
Total reflections measured		23414
Symmetry independent reflections		3010
R_{int}		0.042
Reflections with $I > 2\sigma(I)$		2854
Reflections used in refinement		2799
Parameters refined; restraints		242; 605
Final	$R(F)$ [$I > 2\sigma(I)$ reflections]	0.0668
	$wR(F^2)$ (all data)	0.0819
Weights		Chebychev polynomial
Goodness of fit		1.017
Absolute structure parameter		-0.02(6)
Final Δ_{max}/σ		0.016
$\Delta\rho$ (max; min) [e Å^{-3}]		0.99; -0.97
$\sigma(d_{(C-C)})$ [Å]		0.006 – 0.009

Supplementary Figure 17: Crystallographic data for the Cu-fragin complex.

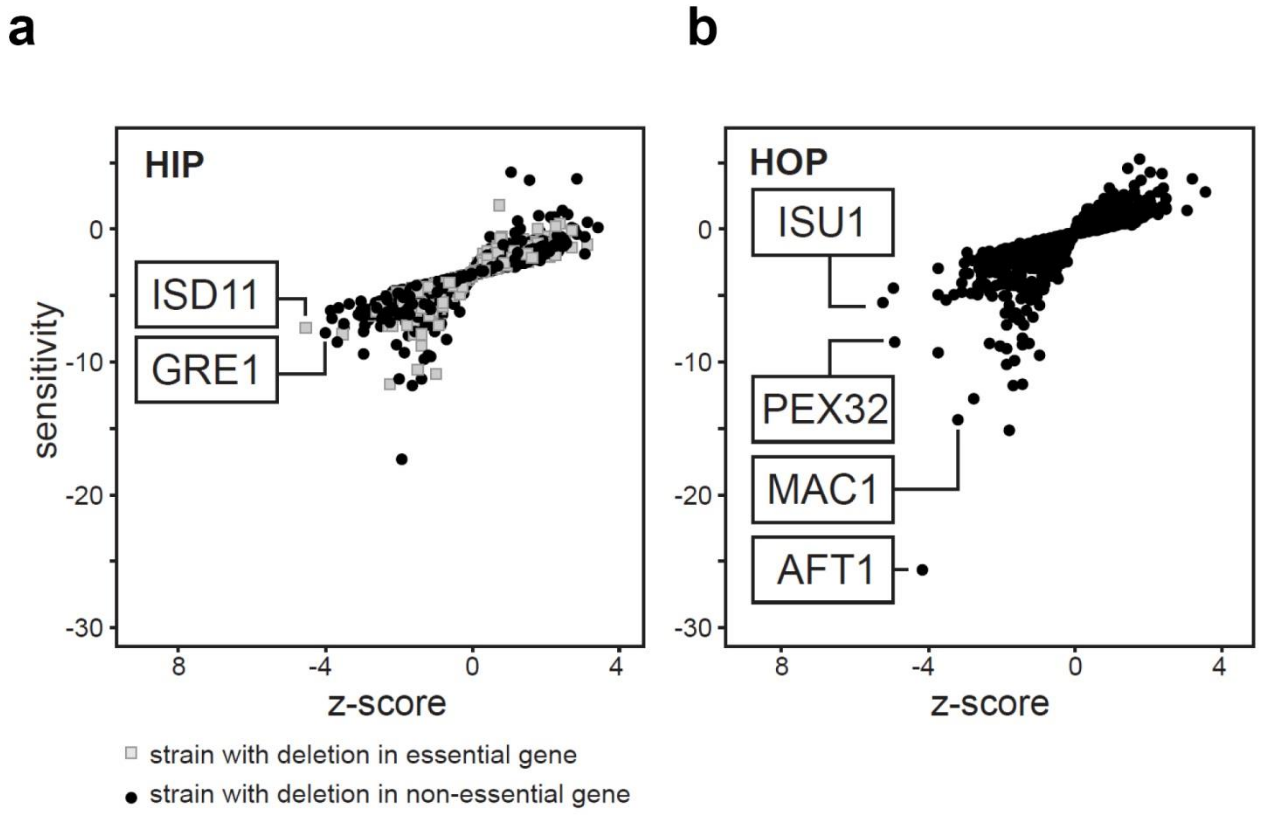

Supplementary Figure 18: HIP-HOP analysis of fragin. **(a)** HIP analysis of fragin in *S. cerevisae*. The best scoring hits (*ISD11*, *GRE1*) are indicated. **(b)** HOP analysis of fragin in *S. cerevisae*. The best scoring hits (*AFT1*, *MAC1*, *PEX32*, *ISU1*) are indicated.

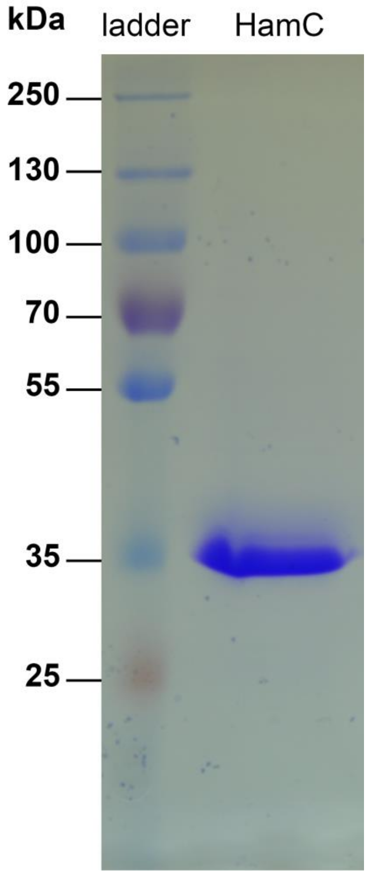

Supplementary Figure 19: SDS-PAGE of heterologously epxressed HamC. HamC was expressed from pQE30 in *E. coli* M15. His-tagged HamC was purified with Ni-NTA agarose beads.

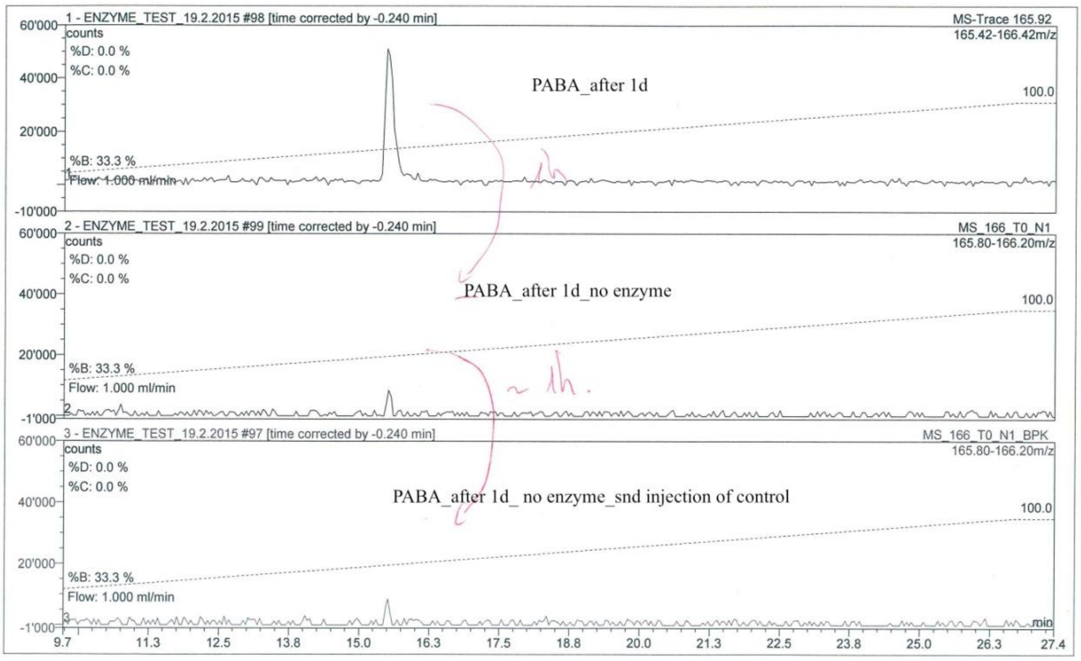

Supplementary Figure 20: Comparison of mass chromatograms in negative mode, extracted at 166 Da (PNBA). The enzymatic conversion of PABA into PNBA using the purified enzyme HamC (top) and the control experiment containing PABA without the enzyme HamC (middle and at the bottom) are displayed. The time between the HPLC-MS analysis of the samples was approximatively 1h.

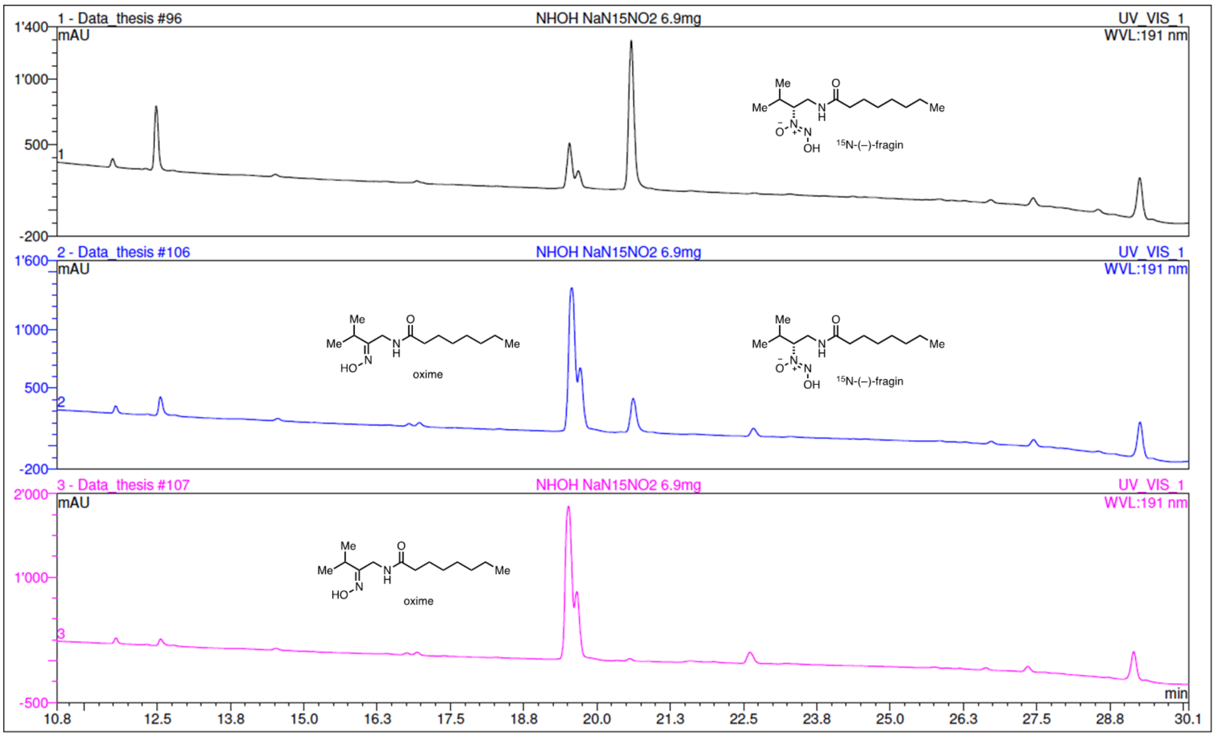

Supplementary Figure 21: HPLC chromatograms of the reaction between $Na^{15}NO_2$ and hydroxylamine. From top to bottom: immediately after preparation, after 24 h and after 44 h.

Supplementary Tables

Supplementary Table 1: Deregulation of the *ham* genes in the published transcriptomes of a pC3-null mutant (ΔpC3) and a CepR quorum sensing mutant (Δ*cepR*) compared to the wild type strain.

Gene	Name	ΔpC3 vs wt	Δ*cepR* vs wt	J2315 ortholog
I35_4188	*hamG*	-5,0	-22,1	bcam0190
I35_4189	*hamF*	-4,9	-12,8	bcam0191
I35_4191	*hamA*	-12,0	-46,8	bcam0192
I35_4192	*hamB*	-12,2	-47,7	bcam0193
I35_4193	*hamC*	-14,5	-52,2	bcam0194
I35_4194	*hamD*	-9,3	-34,0	bcam0195
I35_4195	*hamE*	-10,4	-37,7	bcam0196

Supplementary Table 2: NMR Spectroscopic data ($CDCl_3$, 500 MHz) of (–)-(*R*)-fragin (**1**).

C/N no.	δ_C, type	δ_H ($^3J_{H-H}$ in Hz)	COSY
**1+1'**	18.9, CH_3	0.90, d (6.7)	2
	19.1, CH_3	1.07, d (6.8)	2
**2**	29.1, CH	2.25 - 2.17, m	1, 3
**3**	78.0, CH	4.20, td (9.2, 3.1)	2, 4a, 4b
**NOH**		11.72, s	
**4**	39.1, CH_2	3.86, ddd (14.4, 6.0, 3.1)	NH, 3, 4b
		3.60, ddd (14.4, 9.4, 6.1)	NH, 3, 4a
**NH**		5.69, s	4a, 4b
**5**	173.7,[a] C		
**6**	36.6, CH_2	2.14, td (7.4, 1.8)	7
**7**	25.7, CH_2	1.61 - 1.52, m	6, 8-11
**8 - 11**	22.7,29.1, 29.3, 31.8 (4xCH_2)	1.31 - 1.23, m	7, 12
**12**	14.2, CH_3	0.87, t (6.8)	8-11

[a] Chemical shift recorded using the synthetic product.

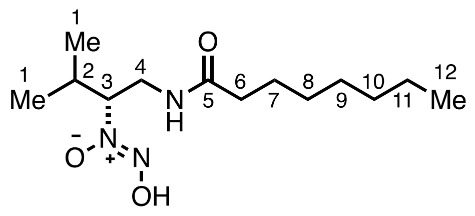

(–)-fragin (**1**)

Supplementary Table 3: NMR Spectroscopic data (500 MHz, CD_3OD) of valdiazen (**2**)

**C no.**	**δ$_C$, type**	**δ$_H$, ($^3J_{H\text{-}H}$ in Hz)**	**δ$_C$, type** of fragin in $CDCl_3$
**1**	19.4, CH_3	0.90, d (6.8)	19.2, CH_3
	19.4, CH_3	1.02, d (6.8)	18.9, CH_3
**2**	29.3, CH	2.12, m	29.1, CH
**3**	82.3, CH	4.04 – 3.96, m	78.0, CH
**4**	61.2, CH_2	3.87 – 3.80, m	39.1, CH_2
		4.00, m	

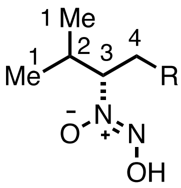

R = OH; (–)-valdiazen (**12**)
R = NHCO(CH_2)$_6$$CH_3$; (–)-fragin (**1**)

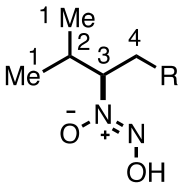

R = OH; (+)-valdiazen (**13**)
R = NHCO(CH_2)$_6$$CH_3$; (+)-fragin (**11**)

Supplementary Table 4: List of the 8 genes with statistically significant increased expression (DESeq analysis, pval < 0.05 and Fold Change > 3) in the chemically complemented *hamD* mutant (H111 Δ*hamD* + 50 µM valdiazen) compared to H111 wild type (wt). The fold change of gene expression is calculated by comparing the chemically complemented *hamD* mutant (H111 Δ*hamD* + 50 µM valdiazen) with the wild type (wt). Locus ID and gene name are according to GenBank file (accession no. HG938370, HG938371, and HG9383729)

Locus ID	Ortholog in J2315	Description	Gene name	fold change H111 Δ*hamD* + 50 µM valdiazen vs wt
I35_0373	BCAL0381	hypothetical protein		3.20E+00
I35_1611	BCAL1700	Outer-membrane receptor for ferri-ornibactin	*orbA*	6.89E+00
I35_2072	BCAL2144	Cytochrome O ubiquinol oxidase subunit II	*cyoA*	3.61E+00
I35_2936	BCAL0729	Nitrogen regulatory protein P-II	*glnB*	3.99E+00
I35_4964	BCAL0544	Dipeptide-binding ABC transporter, periplasmic substrate-binding component (TC 3.A.1.5.2)	*dppA*	4.59E+01
I35_4990	BCAM1143	Hydrolase, alpha/beta fold family		5.79E+00
I35_5466	BCAM1606	Electron transfer flavoprotein, alpha subunit		3.25E+00
I35_7641		transcriptional regulator		3.10E+00

Supplementary Table 5: qRT-PCR results for validation of transcriptomics data

Locus ID	Gene Name	Description	Fold change H111 Δ*hamD* vs H111 wild type	Fold change H111 Δ*hamD* + 50 µM valdiazen vs H111 Δ*hamD*
I35_2532	*ilvD*	Dihydroxy-acid dehydratase	-14.3	-37
I35_6524	*hmuT*	Periplasmic hemin-binding protein	4.61	2.9
I35_4193	*hamC*	*p*-aminobenzoate *N*-oxygenase; AurF	-42	-106
I35_4188	*hamG*	Aminotransferase class III	-33.4	-6.09
I35_2881	*leuA*	2-isopropylmalate synthase	-2.8	-3.32

Supplementary Table 6: Bacterial strains and plasmids used in this study

Strain or plasmid	Characteristics / Information	Source/Reference
Burkholderia cenocepacia		
H111	CF isolate, (Germany)	[3]
H111 Δ*cepI*	Unmarked *cepI* deletion mutant	[4]
H111 Δ*hamA*	Unmarked *hamA* deletion mutant	This study
H111 Δ*hamB*	Unmarked *hamB* deletion mutant	This study
H111 Δ*hamC*	Unmarked *hamC* deletion mutant	This study
H111 Δ*hamD*	Unmarked *hamD* deletion mutant	This study
H111 Δ*hamE*	Unmarked *hamE* deletion mutant	This study
H111 Δ*hamF*	Unmarked *hamF* deletion mutant	This study
H111 *hamG*::pSH	hamG interrupted by pSHAFT2Gm insertion	This study
H111 ΔpC3	H111 after loss of megaplasmid pC3	[5]
Escherichia coli		
Top 10	F- *mcrA*Δ(*mrr-hsdRMS-mcrBC*) ɸ80*lacZ*ΔM15 Δ*lacX*74 nupG recA1 araD139 Δ(ara-leu)$_{7697}$ *galE*15 *galK*16 *rpsL*(Str^R) *endA*1 λ-	Invitrogen
CC118 λpir	Δ(*ara,leu*)$_{7697}$*araD*139 Δ*lacX*74 *galEgalKphoA*20 *thi*-1*rpsErpoB*(RF^R) *argE*(am) *recA*1λpir$^+$	[6]
MC1061	*hsdR araD*139 Δ(*ara-leu*)$_{7697}$ Δ*lacX*74 *galUgalKrpsL* (Sm^R)	[7]
SY327	*araD*Δ(*lac pro*) *argE*(am) *recA*56 Rif^R *nalA*λpir	[8]
DH5α	F$^-$ɸ80Δ*lacZ*ΔM15Δ(*lacZYA-argF*)$_{U169}$ *recA*1*endA*1*hsdR*17 (r_K^-, m_K^+)*supE*44*thi*-1 *relA*1 *gyrA*96	[9]
ATCC25922	Recomended reference strain for antibiotic susceptibility testing	ATCC®
K-12	F$^-$ λ$^-$ rph-1 *ilvG- rfb*-50; MG1655	Eberl lab
S17	*thi recA pro hsdR$^-$ hsdM$^+$* RP4-2-Tc::Mu-Km::Tn7	[10]
HB101	*recA thi pro leu hsd M$^+$* ; Sm^R	[11]
M15	Nal^S, Str^S, Rif^S, Thi$^-$, Lac$^-$, Ara$^+$, Gal$^+$, Mtl$^-$, F$^-$, RecA$^+$, Uvr$^+$, Lon$^+$	Qiagen
**Other bacteria**		
Klebsiella oxytoca 867	wild type	Eberl lab
Bacillus cereus ATTC10876	wild type	ATCC®
Bacillus thuringiensis 1365	wild type	Eberl lab
Staphylococcus aureus DSM 20235	wild type	DSM
Pseudomonas syringae pv. syringae B728a	Bean pathogen; Rif^R	[12]
Chromobacterium violaceum CV0	wild type	Eberl lab

Strain or plasmid (continued)	Characteristics / Information (continued)	Source/Reference (continued)
**Yeast**		
Saccharomyces cerevisae BY4741	MATa his3Δ1 leu2Δ0 met15Δ0 ura3Δ0	Grossniklaus lab, Department of Microbial and Plant Science (University of Zurich)
**Fungus**		
Fusarium solani strain DS185	wild type	Phytopathology Group, Institute of Plant Sciences (Federal Institute of Technology, Zurich, Switzerland)
**Plasmids**		
pBBR	pBBR1MCS2 ; expression vector	[13]
pSU11	vector for *lacZ* fusion	[14]
pEX ::FRT	pEX18TP containinga FRT site ; Gm^R	Eberl lab
pSHAFT ::FRT	pUTmini-Tn5Cm with deleted BglII fragment; contains the *tnp* gene and I end of mini-Tn5; containig a FRT site ; Cm^R	Eberl lab
pBBR5-FLP	pBBR1MCS5 encoding flippase	[5]
pSHAFT2Gm	pUTmini-Tn5Cm with deleted BglII fragment; contains the *tnp* gene and I end of mini-Tn5; Gm^R	[15]
pDAI-SceI	pDAI17 carrying the I-SceI gene	[16]
pGPI-SceI	pGPI-SceIfrom 13 withTpR exchanged to GmR	Eberl lab
pRK600	RK2-*mob*$^+$ RK2-*tra*$^+$, *ori*ColE1	[17]
pQE30	pQE-30	Qiagen
pBBR*hamA*	pBBR1MCS2 expressing *hamA*	This study
pBBR*hamB*	pBBR1MCS2 expressing *hamB*	This study
pBBR*hamC*	pBBR1MCS2 expressing *hamC*	This study
pBBR*hamD*	pBBR1MCS2 expressing *hamD*	This study
pBBR*hamE*	pBBR1MCS2 expressing *hamE*	This study
pBBR*hamF*	pBBR1MCS2 expressing *hamF*	This study

Supplementary References

1. Boeijen, A., van Ameijde, J. & Liskamp, R.M. Solid-phase synthesis of oligourea peptidomimetics employing the Fmoc protection strategy. *J. Org. Chem.* **66**, 8454-8462 (2001).
2. Breuning, M., Hauser, T. & Tanzer, E.M. A novel one-pot procedure for the stereoselective synthesis of alpha-hydroxy esters from ortho esters. *Org. Lett.* **11**, 4032-4035 (2009).
3. Gotschlich, A. et al. Synthesis of multiple *N*-acylhomoserine lactones is wide-spread among the members of the *Burkholderia cepacia* complex. *Syst. Appl. Microbiol.* **24**, 1-14 (2001).
4. Schmid, N. et al. The AHL- and BDSF-dependent quorum sensing systems control specific and overlapping sets of genes in *Burkholderia cenocepacia* H111. *PLoS One* **7**, e49966 (2012).
5. Agnoli, K. et al. Exposing the third chromosome of *Burkholderia cepacia* complex strains as a virulence plasmid. *Mol. Microbiol.* **83**, 362-378 (2012).
6. Herrero, M., de Lorenzo, V. & Timmis, K.N. Transposon vectors containing non-antibiotic resistance selection markers for cloning and stable chromosomal insertion of foreign genes in Gram-negative bacteria. *J. Bacteriol.* **172**, 6557-6567 (1990).
7. Casadaban, M.J. & Cohen, S.N. Analysis of gene control signals by DNA fusion and cloning in *Escherichia coli*. *J. Mol. Biol.* **138**, 179-207 (1980).
8. Miller, V.L. & Mekalanos, J.J. A novel suicide vector and its use in construction of insertion mutations: osmoregulation of outer membrane proteins and virulence determinants in *Vibrio cholerae* requires *toxR*. *J. Bacteriol.* **170**, 2575-2583 (1988).
9. Hanahan, D. Studies on transformation of *Escherichia coli* with plasmids. *J. Mol. Biol.* **166**, 557-580 (1983).
10. Simon, R., Priefer, U. & Puhler, A. A Broad Host Range Mobilization system for in vivo genetic engineering: Transposon mutagenesis in Gram negative bacteria. *Nat. Biotech.* **1**, 784-791 (1983).
11. Boyer, H.W. & Roulland-Dussoix, D. A complementation analysis of the restriction and modification of DNA in Escherichia coli. *J. Mol. Biol.* **41**, 459-472 (1969).
12. Feil, H. et al. Comparison of the complete genome sequences of *Pseudomonas syringae pv. syringae* B728a and *pv. tomato* DC3000. *Proc. Natl. Acad. Sci. U S A* **102**, 11064-11069 (2005).
13. Kovach, M.E. et al. Four new derivatives of the broad-host-range cloning vector pBBR1MCS, carrying different antibiotic-resistance cassettes. *Gene* **166**, 175-176 (1995).
14. O'Grady, E.P., Viteri, D.F., Malott, R.J. & Sokol, P.A. Reciprocal regulation by the CepIR and CciIR quorum sensing systems in *Burkholderia cenocepacia*. *BMC Genomics* **10**, 441 (2009).
15. Carcamo-Oyarce, G., Lumjiaktase, P., Kummerli, R. & Eberl, L. Quorum sensing triggers the stochastic escape of individual cells from *Pseudomonas putida* biofilms. *Nat. Commun.* **6**, 5945 (2015).
16. Flannagan, R.S., Linn, T. & Valvano, M.A. A system for the construction of targeted unmarked gene deletions in the genus *Burkholderia*. *Environ. Microbiol.* **10**, 1652-1660 (2008).
17. Kessler, B., de Lorenzo, V. & Timmis, K.N. A general system to integrate *lacZ* fusions into the chromosomes of gram-negative eubacteria: regulation of the Pm promoter of the TOL plasmid studied with all controlling elements in monocopy. *Mol. Gen. Genet.* **233**, 293-301 (1992).
