# Supplementary material for: Biosynthesis of fragin is controlled by a novel quorum sensing signal
Source: Nat Commun. 2018 Mar 30;9:1297. doi: 10.1038/s41467-018-03690-2 (PMC5878181; doi:10.1038/s41467-018-03690-2)
Supplement: Supplementary file 3 — Description of Additional Supplementary Files(PDF 246 kb) [file 41467_2018_3690_MOESM3_ESM.pdf]

Description of Additional Supplementary Files

File Name: Supplementary Data 1
Description: Homologs of the B. cenocepacia H111 ham genes in bacteria.

File Name: Supplementary Data 2
Description: Genes down-regulated in the *hamD* mutant.

File Name: Supplementary Data 3
Description: Genes up-regulated in the *hamD* mutant.

File Name: Supplementary Data 4
Description: Differentially regulated genes in the *hamD* mutant in the presence of 50 µM valdiazen.

File Name: Supplementary Data 5
Description: Oligonucleotides used in this study.
